# Long-propagating ghost phonon polaritons enabled by selective mode excitation

**DOI:** 10.1038/s41377-025-01925-8

**Published:** 2025-07-26

**Authors:** Manuka Suriyage, Qingyi Zhou, Hao Qin, Xueqian Sun, Zhuoyuan Lu, Stefan A. Maier, Zongfu Yu, Yuerui Lu

**Affiliations:** 1 School of Engineering, College of Engineering, Computing & Cybernetics, the Australian National University, Canberra, ACT 2601 Australia; 2 Department of Electrical and Computer Engineering, University of Wisconsin-Madison, Madison, WI 53706 USA; 3 Department of Physics, Imperial College London, London, SW7 2AZ UK; 4 ARC Centre of Excellence in Quantum Computation and Communication Technology ANU node, Canberra, ACT 2601 Australia; 5 School of Physics and Astronomy, Monash University Clayton Campus, Melbourne, VIC 3800 Australia

**Keywords:** Polaritons, Metamaterials

## Abstract

The ability to precisely control the excitation of phonon polaritons (PhPs) provides unique opportunities for various nanophotonic applications, such as on-chip optical communication, quantum information processing, and controlled thermal radiation. Recently, ghost hyperbolic phonon polaritons (g-HPs) have been discovered, which exhibit in-plane hyperbolic dispersion on the surface and oblique wavefronts in the bulk. These g-HPs exhibit long-range, ray-like propagation, which is highly desirable. However, selective excitation of polaritonic modes and flexible control over the directionality of g-HPs remains an open problem. In this work, we experimentally demonstrate that changing the shape of the launching micro/nano antenna allows for control over the polariton mode excitation. Using a single asymmetric triangular gold antenna fabricated on a calcite crystal surface, we showcase highly directional g-HP excitation through selectively exciting desirable polariton modes. Our near-field imaging experiments verify that the g-HP excited by the triangular antenna can propagate over 80 microns, which is consistent with our numerical predictions. Overall, by combining g-HP theory with structural engineering, our work has further developed the potential of such anisotropic materials, enabling unexpected control over g-HPs, thus opening opportunities for various applications in mid-IR optoelectronics.

## Abstract (web summary)

Directional excitation of ghost hyperbolic phonon polaritons is achieved using an asymmetric antenna, enabling long-range propagation and tunable control for mid-IR nanophotonic applications.

## Graphical abstract

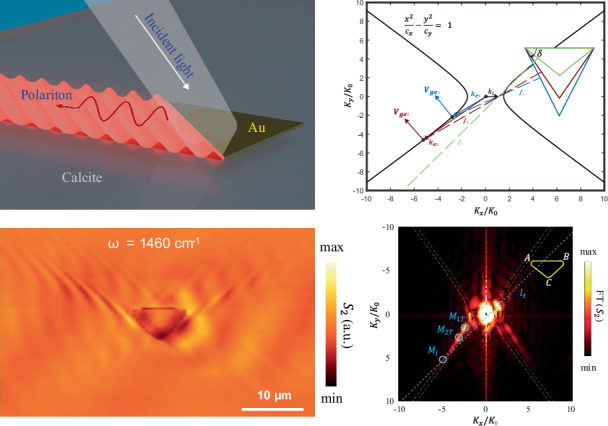

## Introduction

Optical anisotropy is a phenomenon where the dielectric permittivity varies in different directions, resulting in polarized light propagating with distinct velocities and wavelengths along different crystal axes^1–3^. In such crystals with strong material anisotropy, a huge difference in the refractive index along orthogonal axes can be observed^4^. In some cases, the permittivity tensor can become negative within a spectral range, allowing for the excitation of surface polaritons^5^. Natural crystals can be used to produce hyperbolic phonon polaritons if the permittivity tensor elements have opposite signs in principal axes ${\varepsilon }_{t}{\varepsilon }_{z} < 0({\varepsilon }_{t}={\varepsilon }_{x}={\varepsilon }_{y}$ denote the in-plane components, ${\varepsilon }_{z}$ denotes the vertical component). In such systems, the isofrequency curve of light is an open hyperbola^5^. Numerous materials supporting both in-plane and out-of-plane hyperbolic polaritons have been investigated^6^. These include van der Waals thin films^2,7^ with a single optical axis, crystallize in hexagonal^8,9^, trigonal^10^, and tetragonal^11^ systems, as well as biaxial materials with two optical axes, found in orthorhombic^12,13^, monoclinic^14^, and triclinic materials. Two main types of hyperbolic polaritons, namely volume-confined hyperbolic polaritons^15^ and surface-confined hyperbolic polaritons^16^, have been investigated^17^. Most recently, g-HPs^18^ were discovered, which exhibits in-plane hyperbolic dispersion on the surface of a polar uniaxial crystal as well as oblique wave fronts in the bulk, similar to recently predicted ghost waves^19^. Due to the oblique crystal lattice and the ability to control the optical axis angle with respect to the material surface (through mechanically cutting and polishing the sample), calcite enables precise control of its polaritonic response.

Control over the excitation and propagation of phonon polaritons allow useful opportunities for many nanophotonic applications^20–26^. The ability to achieve selective excitation with high directionality and long-propagating PhPs is of particular interest. Highly directional propagation of PhPs is crucial for applications such as optical communication^27^, quantum information processing^28^, coupling between quantum emitters^29^ and heat management^30,31^. In addition, long propagation distance is highly desirable for achieving high signal-to-noise ratio and low-loss transmission. However, the current state-of-the-art techniques face significant limitations in achieving both highly directional and long-propagating PhPs simultaneously^32–34^. For example, the most recent study, which achieved asymmetric propagation by manipulating the in-plane direction of the incident wave^35^, demonstrated directionality but did not achieve long propagation distances.

In this paper, we present a novel approach to achieve selective mode excitation with highly directional and long-propagating PhPs by utilizing an asymmetric micro/nano antenna on a calcite surface. We demonstrate that the directionality and propagation length of PhPs can be flexibly controlled by varying the antenna’s shape and orientation. The microstructure of the triangular shaped antenna is analyzed deeply to provide a technique to selectively excite the polariton modes. By combining our designed system with scattering-type scanning near field optical microscope (s-SNOM)^36–38^, our near-field imaging experiments reveal that the PhPs excited by the triangular antenna can propagate over a very long distance (>80 µm), much longer than the results reported in literatures^12,18^. This breakthrough is achieved by combining g-HP theory with structural engineering, which allows us to excite certain g-HP modes and overcome the limitations of obtaining desired polariton properties such as high directionality and larger propagation lengths. Overall, our work provides new insights into the underlying physics of PhPs and opens opportunities for various applications. The technique introduced here is general and can be extended to other anisotropic materials as well as other types of polariton modes.

This paper is structured as follows: initially, we discuss the innovative design strategies employed to excite highly directional g-HPs, achieved through the utilization of variously shaped antennas. Following this, we discuss edge-assisted mode selection by considering various shapes and orientations of triangle antennas. After that, we demonstrate the ability of the newly developed micro antennas to create considerably longer g-HPs. Finally, we conclude with a comparison of similar work highlighting the uniqueness of our work and outlook.

## Results

### Shape-dependent micro/nano antennas for asymmetric g-HP excitation

**Fig. 1:**
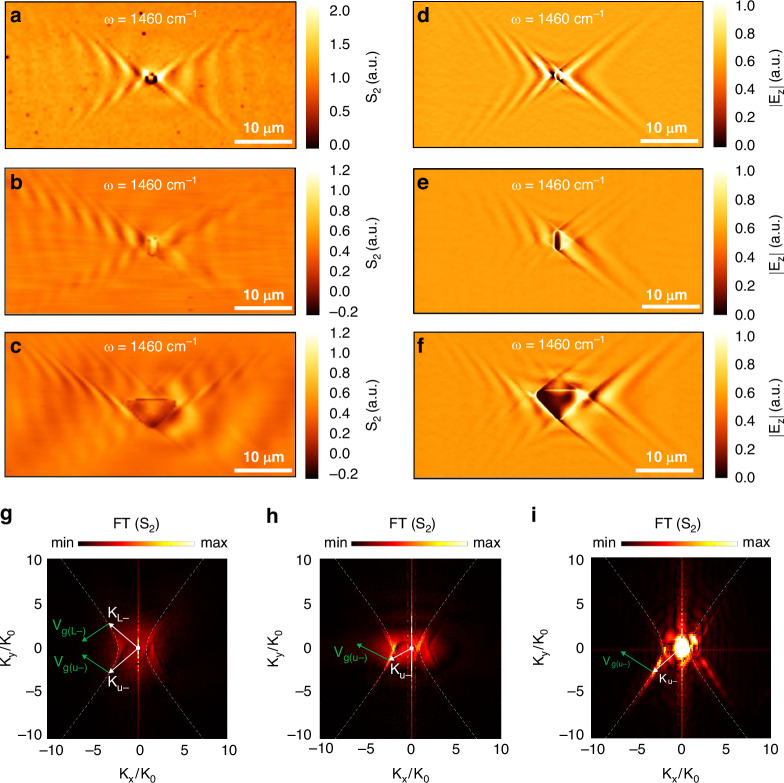
**Shape-Dependent Micro/Nano Antennas for Asymmetric g-HP Excitation.** **a**–**c** Experimental near-field images of antenna-launched (disk, rectangle, triangle) g-HPs at the illumination frequency ${\rm{\omega }}=1460{{\rm{cm}}}^{-1}$. **d**–**f** Simulated near-field images of antenna-launched (disk, rectangle, triangle) g-HPs. **g**–**i** Absolute value of the Fourier transform of the images shown in **a**–**c**, the bright spots represent the modes excited, and the white arrows indicate the wave vectors (momenta) of the antenna-launched g-HPs. Green arrows indicate the energy flow direction/group velocity V_g,(i)_ of the respective polaritonic branch. White dotted curve lines represent the IFC (left and right)

The directionality of a polariton can be crucial for various applications^39,40^ where the direction of the electromagnetic flow at nanoscale becomes important. Based on previous studies this has been achieved through grating diffraction which enables the propagation of the polariton only on one side of the grating^40,41^, via topological transition in background surrounding media such as photonic crystals^42,43^ and hybrid plasmonic structures^14^ and most recently through polarized dipoles^35,44^ and polaritonic crystals^45^. While the grating technique is widely used in photonics, the resulting unidirectional polaritons generated through gratings differ significantly from the wavefronts launched by a point source, such as a nano antenna^40^. But the most recent discovery of asymmetric propagation involves the utilization of a small disk antenna with varying in-plane polarizations, achieved by manipulating the in plane direction of incident light^35,44^. This is an effective way to break the symmetry of the g-HPs excitation. However, by utilizing a disk antenna, multiple g-HP modes with different in-plane wave vectors are excited simultaneously. On the other hand, the technique demonstrated in this paper can selectively excite certain g-HP modes with a given in-plane wave vector, therefore providing much better controllability. Through our experimental demonstration, we utilized different nano/micro antenna shapes to produce highly directional excitations of g-HPs. The motivation comes from the fact that while the calcite surface supports g-HP modes propagating in different directions, it is possible to excite certain modes while suppressing others by merely adjusting the antenna shape. Therefore, by introducing an asymmetric triangular antenna, we demonstrate that we can strongly excite specific modes, leading to a radiation pattern with a desired high directivity. More specifically, three different shapes of antennas were fabricated on the calcite sample (see methods and fabrication section) and the real space imaging of the g-HP excitation was performed using a s-SNOM. Often PhPs are launched more efficiently by the s-SNOM tip^40^ but our experimental observations (Fig. 1a–c) match perfectly with the simulation results for the g-HPs (Fig. 1d–f; generated without considering the s-SNOM tip; Supporting Information Note 2) confirming the absence of tip-launched PhPs in any of our experimental results. We fabricated gold antennas in the shape of disk (diameter D = 1.5 µm; Supplementary Fig. S3b), rectangle (H × W = 3.2 × 1.4 µm; Supplementary Fig. S3e), and triangle (H × L = 4.9 × 8.4 µm; Supplementary Fig. S3h) to demonstrate the controllability of the highly directional propagation of the g-HPs on calcite. The antenna concentrates the 60-degree oblique incident illumination (at 1460 cm^−1^) into a localized hotspot or multiple hotspots depending on the design of the antenna (see Supplementary Figs. S2 and S3), which launches the g-HP. The U and L polariton branches (defined in Supporting information note 3) exhibit varying intensities that demonstrate the selective mode excitation of the polaritons produced by different shaped antennas.

The directionality of the polariton waves was initially confirmed based on the calculated β - directionality constant (Eq. (1)) values and it is further justified by analyzing the FFT results of the respective polaritons.

1 $$\beta =\left|\frac{{\rm{amplitude}}\; {\rm{of}}\; {\rm{the}}\; {\rm{upper}}\; {\rm{polariton}}\; {\rm{branch}}}{{\rm{amplitide}}\; {\rm{of}}\; {\rm{the}}\; {\rm{lower}}\; {\rm{polariton}}\; {\rm{branch}}}\right|$$

The triangle shape shows the highest directionality with a β value of 4.35, meaning that the excited U- branch of the g-HP is more than 4 times stronger than the L- branch. In contrast, the disk exhibits the lowest directionality with a β value of 1.05, meaning that the intensity of U- and L- branches of the g-HPs is almost identical. Based on the relationship in (Supplementary Eq. (S11)), the directionality of the g-HP increases from disk, rectangle to the triangle-shaped antenna. The numerical simulation results (Fig. 1d–f) show similar results to experimental measurements, and the directionality varies similarly based on the calculated β values and FFT results.

It is evident that the shape of the micro/nano antenna can break the symmetry of the propagating polariton waves. Polariton’s group velocity ${V}_{g(i)}$ (where i = U+, U−, L+, L−; L: lower branch, U: upper branch) represents the energy flow direction. For a disk-shaped antenna (Fig. 1a) all four polariton branches are visible in the experimental results and the FT shown in Fig. 1g shows that the disk antenna is capable of exciting multiple modes in all 4 branches (U+, U−, L+, L−) with different coupling efficiencies. Two modes (highest k modes excited by the disk antenna) are visualized using the k vectors (${K}_{L-},{K}_{U-}$) in the FT image with the corresponding group velocities. The brightness of those bright spots in k space represents the intensities of the polaritonic branch with that specific polariton mode. Figure 1h is the FFT result for the polariton generated by the rectangular antenna (Fig. 1b). Here the change of shape has modulated the intensity of the U- branch and it is extremely high according to the brightness of the spot that represents, ${K}_{U-}$ vector in panel h. But still, it is not as efficient as a triangle shape antenna to excite modes selectively. The FFT result (Fig. 1i) of the polariton generated by the triangular antenna (Fig. 1c) clearly showcases two bright regions with one having discrete extremely bright spots (U- branch). Therefore, the triangular shaped antenna is only exciting a collection of modes that generated two branches with the wavevectors, ${K}_{U-}$ and, ${K}_{U+}$ and the ${K}_{U-}$ is higher in intensity compared to the ${K}_{U+}$ polaritonic branch.

### Edge assisted selective mode excitation

**Fig. 2:**
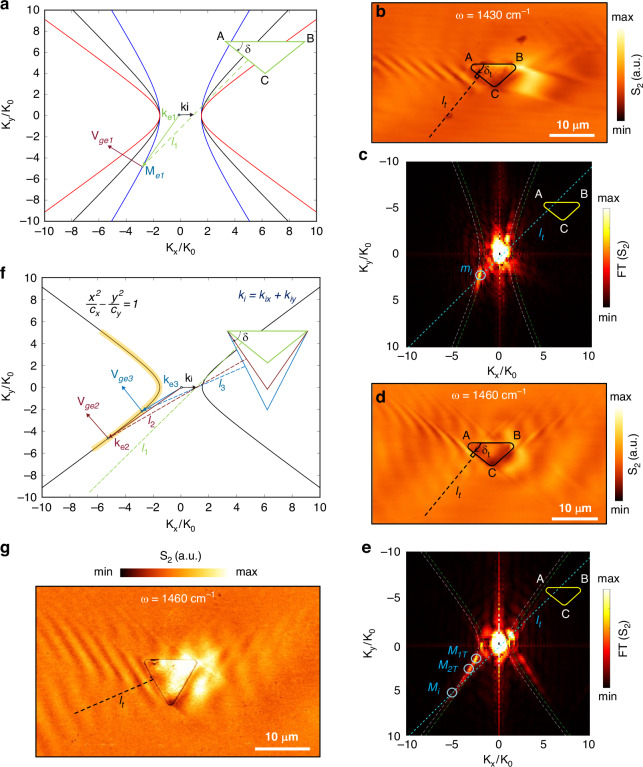
**Edge assisted mode selection.** **a** Schematic illustration of the edge assisted mode selection by a triangular antenna for different excitation frequencies. The blue, black, red solid line represents the IFC for calcite at ${{{1430}\,{cm}}^{-1},{1460}\,{cm}}^{-1},{{1490}\,{cm}}^{-1}$. ${l}_{1}$ is the line normal to the AC edge of the triangle. ${M}_{e1}$ is the mode at the intersection of IFC and ${l}_{1}$ lines. ${K}_{e1}$ represent the corresponding wave vectors for mode ${M}_{e1}$. ${k}_{i}$ represent the wave vector for the incident wave. **b** Near-field amplitude images of a triangular antenna-launched g-HPs at the illumination frequency ${\rm{\omega }}=1430\,{{\rm{cm}}}^{-1}$. **c** Absolute value of the Fourier transforms of the images shown in b. ${m}_{i}$ is the intersection point of line ${l}_{t}$ and the IFC at ${\rm{\omega }}=1430\,{{\rm{cm}}}^{-1}$. **d** Near-field amplitude images of a triangular antenna-launched g-HPs at the illumination frequency ${\rm{\omega }}=1460\,{{\rm{cm}}}^{-1}$. Triangle inner angle ${\delta }_{t}={43}^{^\circ }$ and ${l}_{t}$ is the normal axis to the AC edge of the triangle. **e** Absolute value of the Fourier transforms of the images shown in d. ${M}_{1t}$ and ${M}_{2t}$ are the antenna excited modes at ${\rm{\omega }}=1460\,{{\rm{cm}}}^{-1}$. ${M}_{i}$ is the intersection point of line ${l}_{t}$ and the IFC at ${\rm{\omega }}=1460\,{{\rm{cm}}}^{-1}$. **f** Schematic illustration of the edge assisted mode selection at the boundary by tuning the size of the triangle antenna. Black solid line represents the IFC for calcite at ${1490\,{cm}}^{-1}$. Green, red, and blue triangles represent three separate triangles with three distinct angles $\delta \,={\delta }_{1},{\delta }_{2},\,{\delta }_{3}$. ${l}_{1},{l}_{2},\,{l}_{3}$ are the three lines normal to the AC edge of each triangle. ${M}_{e2}$, ${M}_{e3}$ are the two modes at the intersection of IFC and ${l}_{2},{l}_{3}$ lines. ${K}_{e2}$ and ${K}_{e3}$ represent the corresponding wave vectors for modes ${M}_{e2}$ and ${M}_{e3}$. **g** Near-field amplitude image antenna-launched g-HPs at the illumination frequency ${\rm{\omega }}=1460\,{{\rm{cm}}}^{-1}$ for a triangular antenna with a larger internal angle ${\delta }_{t}={60}^{^\circ }$

Controlling the properties of polaritons is essential in various applications, such as sensing^46^, communication, and optoelectronics^23^. This is normally achieved by adjusting the size and refractive index of the polariton system^17,47^, modifying the coupling strength^21,48^, and using patterned structures^17,18,38^. Recent research has focused on investigating negative reflection^49^ and negative refraction^50,51^ in hyperbolic media but has not demonstrated the capability for selective excitation. The excitation frequency of the illumination source determines important parameters of ghost polariton, particularly the directionality angle α of the hyperbolic polariton (Supplementary Fig. S6). Our simulation and experimental results show that α increases w.r.t to the illumination frequency, but the open angle of the excited g-HP from a triangle antenna is not consistent with the excited g-HPs from a disk antenna. As schematically illustrated in Fig. 2a, a triangular-shaped antenna with an internal angle of $\delta$ can selectively excite a g-HP mode if a mode on the IFC intersects the line ${l}_{1}$ which is perpendicular to the AC edge. The schematic displays three IFCs, and for excitation at $\omega =1430\,{{cm}}^{-1}$ the triangle with this specific angle can exclusively excite the mode aligned perpendicularly to the edge.

We demonstrated this using our s-SNOM measurements as illustrated in Fig. 2b–e. When the triangle antenna is excited at $\omega =1430\,{{cm}}^{-1}$ as in Fig. 2b we observe parallel polaritonic fringes. The FFT result displayed in Fig. 2c reveals a solitary bright spot, indicating the excitation of a single polariton mode. As anticipated, this mode ${m}_{i}$ corresponds to the intersection point of the respective IFC at $\omega =1430\,{{cm}}^{-1}$ and line ${l}_{t}$, underscoring our successful realization of selective excitation of polariton modes through the utilization of a physical edge. However, when the same triangular antenna is excited at $\omega =1460\,{{cm}}^{-1}$ we can see directional propagation, but the triangle is exciting multiple modes, as evidenced by the presence of numerous bright spots in the FT image in Fig. 2e. The two most prominent excited modes are labeled as ${M}_{1T}$ and ${M}_{2T}$, corresponding to mode ${M}_{i}$ which is positioned on the IFC where it intersects with line ${l}_{t}$ (perpendicular axis to the AC edge of the triangle). Thus, at $\omega =1460\,{{cm}}^{-1}$ the triangular shape facilitates the coupling of multiple modes with high directionality in the U-branch rather than exciting a single edge assisted mode. Although the IFC supports an infinite number of modes, only a few discrete modes are efficiently coupled by the antenna to excite polaritons, depending on the curvatures of the three vertices. The orange-shaded region in Fig. 2f highlights that this coupling is limited and can be tailored by adjusting the curvature and size of the antenna. The AC edge of the antenna in Fig. 2d should be able to selectively excite a high k mode (${M}_{i}$) as shown in Fig. 2e at $\omega =1460\,{{cm}}^{-1}$ but the curved edges and the confinement of the antenna is not enough to excite that mode. So, we believe that the triangular antenna’s curved edge triggers a specific set of polaritonic modes towards the U- branch and, simultaneously, the angled physical edge of the triangular antenna facilitates high coupling efficiency to the polariton modes which has wave vectors perpendicular to the edge. Edge-assisted mode selection has minimal impact at larger wavenumbers because the angle of the antenna’s physical edge is not sufficient unless the angle is increased a lot to couple or scatter modes with wave vectors that are perpendicular to the edge (Supplementary Fig. S6c, f).

However, if we want to use a single excitation frequency and do selective mode excitations, we can now change the internal angle of the triangle antenna as shown in the schematic of Fig. 2f. If the curved edges (A and C) of the blue triangle in the schematic with an internal angle ${\delta }_{3}$ can excite the mode ${M}_{e3}$, then the physical edge enhances the coupling efficiency of that specific mode. When the angle is reduced to ${\delta }_{2}$ for the edge to enhance coupling efficiency, the vertices should efficiently excite mode ${M}_{e2}$. Thus, as the triangle’s internal angle δ decreases, the edge-assisted coupling mode will correspond to a higher k vector. These wave vectors can be efficiently coupled by adjusting the antenna’s size in relation to the curvatures and edge lengths. But beyond a certain critical angle the edge will not be able to support mode selection as demonstrated through the smallest triangle with an internal angle of ${\delta }_{1}$.

To demonstrate this experimentally we fabricated a triangle with a larger internal angle and excited it at $\omega =1460\,{{cm}}^{-1}$ as shown in Fig. 2g and now the triangle can selectively excite a single mode with propagating parallel fringes. Therefore, by adjusting the internal angle of triangle δ, we can alter the inclination of the AC physical edge of the triangle shape, offering a means to regulate selective mode excitation.

**Fig. 3:**
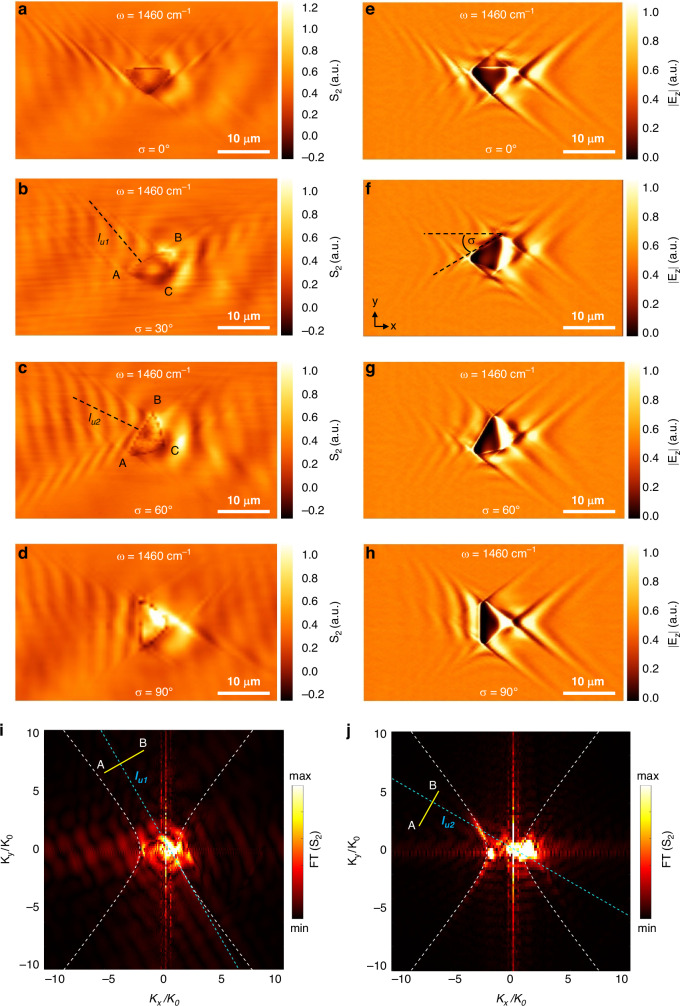
**Edge assisted mode selection based on the rotational angle (σ) of the micro/nano antenna.** **a**–**d** Experimental near-field images of antenna-launched g-HPs for the rotational angles σ = 0^°^, 30^°^, 60^°^, 90^°^. ${l}_{u1}$ and ${l}_{u2}$ are the perpendicular axis to the BA physical edge of the triangles with rotational angles σ = 30^°^, 60^°^. **e**–**h** Simulated near-field images of antenna-launched g-HPs, for the rotational angles σ = 0^°^, 30^°^, 60^°^, 90^°^. **i**, **j** Absolute value of the Fourier transforms of the images (**b**) and (**c**). Yellow AB line represents the AB physical edge of the triangle

Then we explored the change of orientation of the micro/nano antenna to modulate the directionality of the polariton waveform generated in both experimental and simulation results. The orientation angle σ of the antenna relative to the crystal axis (Fig. 3f; angle w.r.t the x axis of the calcite sample surface) was changed to govern the diffraction and the directionality of the g-HP. Figure 3a–d illustrates the measured s-SNOM images of the g-HPs with antenna orientation angles σ = ${0}^{^\circ }$, ${30}^{^\circ }$, ${60}^{^\circ }$ and ${90}^{^\circ }$, respectively. All our experimental near-field images show excellent agreement with the simulation results (Fig. 3e–h), supporting the validity of the observed directionality in experimental measurements. Here the position of the vertices and the angle of the physical edge of the triangular antenna changes resulting in selective excitation of polaritonic mode as explained before for the non-rotated triangle.

Similar to how the AC physical edge facilitated mode selection in the U- branch, we demonstrate the capability to excite modes in the L- polariton branches by rotating the triangle along the crystal axis. As Illustrated in Fig. 3, when the triangle is rotated with respect to the x-axis at a 60-degree angle the L- polariton branch is excited, and polariton fringes emerge parallel to the BA (Fig. 3c) physical edge. This phenomenon arises from the interplay between polaritons excited by the curved vertex (B) and the physical edge BA, maintaining the parallel fringes to the edge unchanged. The FT result in Fig. 3j justifies this, with a bright spot evident at the intersection points of the IFC and the line ${l}_{u2}$ (the perpendicular axis to the BA edge of a 60-degree rotated triangle antenna). However, at a lower rotational angle, as depicted in Fig. 3b (30 degrees), the edge fails to support the excited modes since ${l}_{u1}$ does not intersect with the respective IFC, as illustrated in Fig. 3i. Therefore, we have demonstrated various methods for antenna design and the control of selective mode excitation, leveraging curved edges, particularly through the technique of edge-assisted mode selection.

### Highly enhanced propagation length of g-HPs through selective mode excitation

The propagation length of phonon polaritons is an important parameter that quantifies the distance over which these hybrid light-matter quasiparticles can propagate before they are absorbed or scattered. A high propagation length is desirable in phonon polaritons for several reasons, such as increased sensitivity, enhanced energy transfer rate, longer range interactions, leading to improved device performance^32,52^. The modulation of phonon polariton length have been achieved through different techniques: modifying material properties, adjusting the strength of light-phonon coupling, reducing scattering and absorption through material selection and engineering techniques^53–56^, and incorporating phonon resonators in the polaritonic structure^25^. But none of these have demonstrated high propagation lengths experimentally through selective mode excitation.

**Fig. 4:**
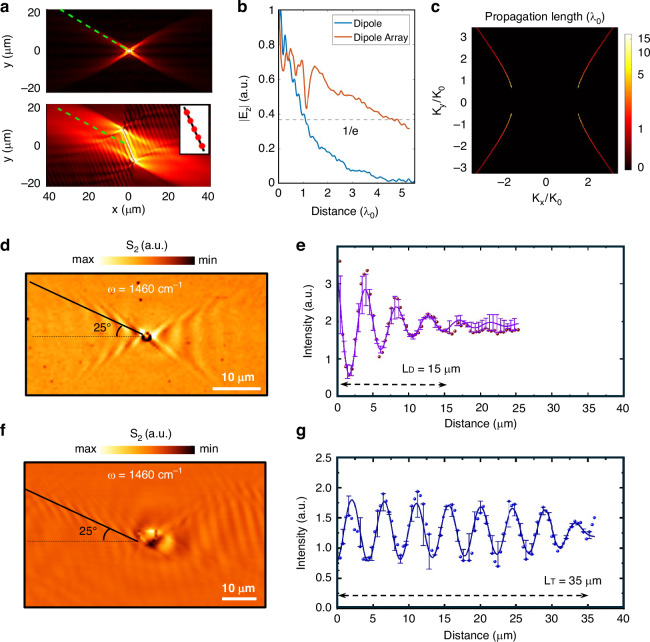
**Highly enhanced propagation length of g-HPs through different shaped micro/nano antennas.** **a** Simulated comparison of g-HP wave excitation by a single dipole and a dipole array consisting of $N=51$ dipoles (interval $d=0.04{\lambda }_{0}$, array orientation matched with the g-HP mode located at ${k}_{x}=-1.604{k}_{0},{k}_{y}=-0.648{k}_{0}$). The above field distributions are calculated at frequency $1460\,{{\rm{cm}}}^{-1}$. All dipole sources are oriented in the $z$ direction. **b** $|{E}_{z}|$ linecuts along green dashed lines in (**a**). **c** Propagation lengths of g-HP modes (calculated at frequency $1460\,{{\rm{cm}}}^{-1}$) on the IFC, visualized in momentum space. The propagation lengths have been normalized using the wavelength ${\lambda }_{0}$ inside vacuum. **d** Experimental near-field result of a disk-shaped nano antenna with a larger scanning area of 50 × 25 µm. **e** Line cut profile data for the disk-shaped nano antennas with an angle of 25^°^ w.r.t the x axis. Dark red balls represent the actual line cut profile data and the purple line is the sine damp fitted function explained in Supplementary Note 5 with a decay constant of $1.579\times {10}^{5}\,{m}^{-1}$. **f** Experimental near-field result of a triangular-shaped micro antenna with an inner angle *δ* = 55^°^ and a scanning area of 70 × 50 µm. **g** Line cut profile with an angle of 25^°^ w.r.t the x axis for the triangle-shaped micro antenna in g. Blue balls represent the actual line cut profile data and the dark blue line is the sine damp fitted function (Eq. (S21)) with a decay constant of $1.44\times {10}^{4}\,{m}^{-1}$. The error bars in e and g represent the fitting uncertainty

In this study, we demonstrate extended propagation lengths by selectively exciting specific low-$k$ g-HP modes. We are mainly focusing on the g-HP modes that propagate at the calcite-air interface. The modes that propagate inside bulky calcite are ignored since they cannot be observed by our s-SNOM system. A small disk antenna, with a sufficiently small diameter, acts as a dipole source^35^ capable of exciting all g-HP modes, including both high-$k$ and low-$k$ modes. However, the emitted energy is distributed among multiple electromagnetic modes^57^, leading to a shorter dipole-dipole interaction range. Therefore we simulated this scenario as is illustrated in Fig. 4a (top panel), where a single dipole oriented along the z-axis excites multiple g-HP modes simultaneously. In contrast, a dipole array with dipoles, spaced closely apart, selectively excites g-HP modes (Fig. 4a, bottom panel). The ${E}_{z}$ field distributions along the dotted lines are plotted in Fig. 4b, revealing that restricting the excitation to specific g-HP modes enhances the propagation length by approximately fourfold. These simulation results substantiate our claim, modifying the polariton excitation method can significantly alter the decay pattern of the resulting $\vec{E}$ field distribution.

We also performed a theoretical analysis and numerically calculated the propagation lengths of all g-HP modes residing on the IFC. The results, normalized to the vacuum wavelength ${\lambda }_{0}$ are visualized in Fig. 4c (details provided in Supporting Information Note 5). It can be seen that for certain low-$k$ modes, the propagation length can become very large ($>\! 10{\lambda }_{0}$). For instance, at 1460 cm^−1^ the predicted propagation length ${L}_{p}$ exceeds $119.5\,\mu {\rm{m}}$ (details regarding the prediction of propagation lengths are provided in Supporting Information Note 5 and 6) for the g-HP mode located at $\left(\mathrm{Re}{k}_{x},\,\mathrm{Re}{k}_{y}\right)=(-1.590{k}_{0},\,0.588{k}_{0})$. If the incident beam can be coupled to that long-propagating mode strongly, it would be possible to observe the existence of this g-HP mode.

Based on our experimental results, the newly designed triangular shape antenna shows longer propagation lengths. This is because the antenna is capable of selectively exciting polariton modes that can propagate an extended distance. Therefore, we can achieve the highest possible propagation length for calcite just by engineering the antenna’s edge orientation to selectively excite certain long propagating polariton mode. Previous research works have shown that most phonon propagation lengths are limited to a few microns^1,2,11,16,58^, while the recently discovered g-HPs showed larger propagation lengths up to 20 microns^18^. In our experimental results we observe much longer propagation lengths exceeding 80 µm (Fig. 4f) for the triangular shape antenna. We can clearly see that the intensity of the polariton wave (Fig. 4g) obtained from the line cuts shown in respective SNOM images (Fig. 4f) for the triangular shape is strong even at 30–35 µm (with an estimated propagation distance of 80 µm with the damping constant), demonstrating the potential of having extended longer polariton wavefronts. The propagation length for the disk (Fig. 4d) matches with previous reports^18^. The propagation length of the disk antenna according to the line cut profile in Fig. 4e with a decay constant of $1.782\,\times {10}^{5}\,{{\rm{m}}}^{-1}$ is around 15 µm. The newly designed rectangle and triangle shaped antennas exhibit much stronger and longer propagations (Fig. 1b, c). The triangular shape yields the longest propagation length, and the size and curvature of the antenna can further control the propagation length of the polariton. We have clearly observed extended propagation distances in the case of three triangular antennas, as depicted in Fig. 4f and Supplementary Fig. S11. In this case we can obtain single mode parallel fringes (parallel to the AC edge) and we can maintain the propagation to larger distances. Although theoretical predictions suggest propagation lengths of up to 119.5 µm, our observations yield a reduced value of 82.3 µm. We attribute this discrepancy to several non-ideal factors: imperfect edge-assisted mode excitation (which introduces short-propagating modes), imperfect mode coupling due to oblique incidence, as well as scattering losses from impurities in the calcite substrate.

## Discussion

In conclusion, our experimental work has demonstrated the remarkable selective mode excitation of g-HPs using an asymmetric launching antenna on a calcite crystal surface. By manipulating the antenna’s shape, orientation, and excitation wavelength we have shown that the g-HP modes can be selectively excited to achieve high directionality and high propagation length. The mode selection can be flexibly controlled by edge-assisted selective mode excitation technique that we demonstrate in the manuscript. Moreover, with the help of s-SNOM system, our near-field imaging experiments have revealed that the g-HP excited by the triangular antenna can propagate over a very long distance (more than 80 µm). All experimental results are consistent compared with simulation results.

Our work not only advances our understanding of the fundamental physics underlying g-HPs, but also offers new opportunities for developing a wide range of nanophotonic applications. By taking advantage of the interplay between structural engineering and g-HP theory, we have proved that anisotropic materials such as calcite have the potential to serve as a good platform for developing next-generation nanophotonic devices. In particular, such highly directional, long-propagating g-HP modes could be valuable for quantum information processing, on-chip optical communication, and high-precision sensing applications. The technique introduced here is general, and can be readily extended to other anisotropic materials. We anticipate that our work will stimulate further research in this rapidly growing field.

## Materials and methods

### Numerical Simulations

We use COMSOL Multiphysics 5.1 software to simulate the electric field distribution of ghost polaritons emitted by gold antenna. Under oblique incidence (p-polarized plane wave, the incident angle is set as ${\varphi =60}^{^\circ }$), the gold antenna will launch polariton wave propagating along the surface of calcite sample. After calculating the scattered electric field using finite element method, the intensity of electric field (recorded at 100 nm above the calcite surface) is plotted and compared with experimental results.

As for the permittivity of calcite crystal, we used a Lorentz oscillator model:

2 $${\varepsilon }_{\perp }={\varepsilon }_{{\infty },1}\left(1+\frac{{\omega }_{{LO},1}^{2}-{\omega }_{{TO},1}^{2}}{{\omega }_{{TO},1}^{2}-{\omega }^{2}-i\omega {\Gamma }_{1}}+\frac{{\omega }_{{LO},2}^{2}-{\omega }_{{TO},2}^{2}}{{\omega }_{{TO},2}^{2}-{\omega }^{2}-i\omega {\Gamma }_{2}}\right)$$

3 $${\varepsilon }_{\parallel }={\varepsilon }_{{\infty },3}\left(1+\frac{{\omega }_{{LO},3}^{2}-{\omega }_{{TO},3}^{2}}{{\omega }_{{TO},3}^{2}-{\omega }^{2}-i\omega {\Gamma }_{3}}\right)$$

The optic axis is initialized to be aligned with y-axis and is then rotated with respect to x-axis by $\theta ={23.3}^{^\circ }$.

The parameter of gold antenna is given as follows: for the “disk” antenna, its radius is 0.75 μm; for the “rectangle” antenna, its length is 3.2 μm, while its width is 1.4 μm; for the “triangle” antenna, its length is 8.4 μm, while its width is 4.9 μm. For all simulations, the thickness of gold antenna is set to be 50 nm. More simulation details can be found in Supplementary Information (Supporting Information Note 1 and 2).

### Sample preparation

We used a commercially available polished calcite substrate (size: 10 mm × 10 mm × 0.5 mm) that was prepared by mechanical cleavage from bulk calcite single crystal (trigonal structure). Electron beam lithography was used to fabricate the gold micro/nano antennas on the calcite substrate. The patterns were written on the resist (ARP: 6200/0.9) spin coated on the substrate (at 4000 rpm for 1 min and baked at 150 °C for 1 min). Cr (5 nm) and Au (45 nm) were deposited using e-beam evaporation and the standard lift off procedure (ZEP remover overnight) was used to finalize the fabrication.

### s-SNOM measurements

A commercially available s-SNOM (from Neaspec) that is based on a tapping mode atomic force microscope (AFM) was used to perform the real-space imaging of the polaritonic patterns. The tip was controlled to maintain a tapping amplitude of 70 nm. The Pt coated AFM tip is illuminated using a quantum cascade laser (QCL) at an angle about ${60}^{^\circ }$ with respect to the substrate surface. The QCL wavelength can be tuned from 1310 cm^−1^ to 1705 cm^−1^.

## Supplementary information

Supplementary Material

## Data Availability

The data and the code that supports the findings of this research are available from the corresponding authors upon reasonable request.
