# Supplementary material for: Long-propagating ghost phonon polaritons enabled by selective mode excitation
Source: Light Sci Appl. 2025 Jul 26;14:254. doi: 10.1038/s41377-025-01925-8 (PMC12296731; doi:10.1038/s41377-025-01925-8)
Supplement: Supplementary file 1 — Supplementary Material [file 41377_2025_1925_MOESM1_ESM.pdf]

Supplementary Information

Long-Propagating Ghost Phonon Polaritons Enabled by Selective Mode Excitation.

Manuka Suriyage [1#], Qingyi Zhou[2#], Hao Qin[1], Xueqian Sun[1], Zhuoyuan Lu[1], Stefan A Maier[4] , Zongfu Yu[2*] and Yuerui Lu[1,3*]

[1]School of Engineering, College of Engineering, Computing & Cybernetics, the Australian National University, Canberra, ACT, 2601, Australia

[2]Department of Electrical and Computer Engineering, University of Wisconsin-Madison, Madison, WI 53706, USA

[3]ARC Centre of Excellence in Quantum Computation and Communication Technology ANU node, Canberra, ACT 2601, Australia.

[4]School of Physics and Astronomy, Monash University Clayton Campus, Melbourne, Victoria 3800, Australia

# Those two authors contribute equally to this work.

* To whom correspondence should be addressed: Yuerui Lu (yuerui.lu@anu.edu.au), Zongfu Yu (zyu54@wisc.edu)

Supporting Information Note 1: Theory of g-HP mode in rotated calcite

In this section, by solving Maxwell's equations in frequency domain[1], we verify the existence of ghost polariton mode at the interface between calcite and vacuum.

Consider the interface between anisotropic calcite crystal ($z < 0$) and vacuum ($z \geq 0$). The optic axis OA of calcite crystal is initialized to be aligned with y-axis. Then the OA is rotated by an angle θ with respect to x-axis. Therefore, the permittivity tensor of calcite substrate can be written as:

$$\bar{\bar{\varepsilon}} = R_x(-\theta) \cdot \begin{pmatrix} \varepsilon_\perp & 0 & 0 \\ 0 & \varepsilon_\parallel & 0 \\ 0 & 0 & \varepsilon_\perp \end{pmatrix} \cdot R_x(\theta), \qquad \text{(S1)}$$

where $R_x(\theta)$ represents the rotation matrix with respect to x-axis. The permittivity tensor can be derived as:

$$\bar{\bar{\varepsilon}} = \begin{pmatrix} \varepsilon_\perp & 0 & 0 \\ 0 & \varepsilon_\parallel \cos^2\theta + \varepsilon_\perp \sin^2\theta & (\varepsilon_\parallel - \varepsilon_\perp)\sin\theta\cos\theta \\ 0 & (\varepsilon_\parallel - \varepsilon_\perp)\sin\theta\cos\theta & \varepsilon_\perp \cos^2\theta + \varepsilon_\parallel \sin^2\theta \end{pmatrix}. \qquad \text{(S2)}$$

Now we are ready to start with the source-free Maxwell's equation. Note that only electric field $\vec{E}$ is kept:

$$\nabla \times \nabla \times \vec{E} - k_0^2 \bar{\bar{\varepsilon}} \cdot \vec{E} = 0, \qquad \text{(S3)}$$

where k_0 denotes the wave vector inside vacuum. We'd like to focus on eigenmodes with $e^{i\vec{k}\cdot\vec{r}}$ dependence. The above Maxwell's equation is thus simplified:

$$\vec{k} \times \vec{k} \times \vec{E} + k_0^2 \bar{\bar{\varepsilon}} \cdot \vec{E} = 0. \qquad \text{(S4)}$$

Denote the components of $\vec{k}$ as $\vec{k} = [k_x, k_y, k_{2z}]$. The fact that this equation has non-zero solution for $\vec{E}$ indicates that the following determinant equals zero:

$$\begin{vmatrix} -k_{2z}^2 - k_y^2 + \varepsilon_\perp k_0^2 & k_x k_y & k_{2z} k_x \\ k_x k_y & -k_{2z}^2 - k_x^2 + k_0^2(\varepsilon_\parallel \cos^2\theta + \varepsilon_\perp \sin^2\theta) & k_{2z} k_y + k_0^2(\varepsilon_\parallel - \varepsilon_\perp)\sin\theta\cos\theta \\ k_{2z} k_x & k_{2z} k_y + k_0^2(\varepsilon_\parallel - \varepsilon_\perp)\sin\theta\cos\theta & -k_x^2 - k_y^2 + k_0^2(\varepsilon_\perp \cos^2\theta + \varepsilon_\parallel \sin^2\theta) \end{vmatrix}. \qquad \text{(S5)}$$

With the help of Wolfram Mathematica 12.3, we conduct a factorization, which leads to two equations:

$$k_x^2 + k_y^2 + k_z^2 = \varepsilon_\perp k_0^2, \qquad \text{(S6)}$$

$$\frac{(k_y \cos\theta + k_{2z}\sin\theta)^2}{\varepsilon_\perp} + \frac{k_x^2 + (k_y \sin\theta - k_{2z}\cos\theta)^2}{\varepsilon_\parallel} = k_0^2. \qquad \text{(S7)}$$

The first equation corresponds to ordinary wave (o-wave), while the second equation corresponds to extraordinary wave (e-wave).

For arbitrary given k_x and k_y, the value of k_{2z} can be derived by solving the quadratic equation:

$$k_{2z} = \frac{-k_y(\varepsilon_\parallel - \varepsilon_\perp)\sin\theta\cos\theta \pm \sqrt{\Delta}}{\varepsilon_\perp \cos^2\theta + \varepsilon_\parallel \sin^2\theta}, \tag{S8}$$

where $\Delta = \varepsilon_\perp[\varepsilon_\parallel k_y^2 + (\varepsilon_\perp \cos^2\theta + \varepsilon_\parallel \sin^2\theta)(k_x^2 - \varepsilon_\parallel k_0^2)]$. When $\Delta < 0$, k_{2z} becomes complex, which means that the extraordinary wave is not only decaying inside calcite, but also shows oblique wavefront. The g-HP modes we want consist of a superposition of both the o-wave and the e-wave. Details regarding how to calculate the field distribution of these g-HP modes numerically have been included in Supporting Information Note 5.

Supporting Information Note 2: Detailed information of numerical simulations

In this section, we introduce more details regarding the numerical simulations presented in the main text. We use COMSOL Multiphysics 5.1 software for all the numerical simulations.

As we've mentioned in the main text, we use a Lorentz oscillator model1 to calculate the permittivity of calcite substrate (Figure S1):

$$\varepsilon_{\perp} = \varepsilon_{\infty,1}\left(1 + \frac{\omega_{LO,1}^2 - \omega_{TO,1}^2}{\omega_{TO,1}^2 - \omega^2 - i\omega\Gamma_1} + \frac{\omega_{LO,2}^2 - \omega_{TO,2}^2}{\omega_{TO,2}^2 - \omega^2 - i\omega\Gamma_2}\right) \tag{S9}$$

$$\varepsilon_{\parallel} = \varepsilon_{\infty,3}\left(1 + \frac{\omega_{LO,3}^2 - \omega_{TO,3}^2}{\omega_{TO,3}^2 - \omega^2 - i\omega\Gamma_3}\right) \tag{S10}$$

The OA is initialized to be aligned with y-axis and is then rotated with respect to x-axis by $\theta = 23.3^{\circ}$.

For Au, permittivity data measured experimentally is used[2]. Inside the wavelength range we're interested in ; Au has a negative permittivity whose absolute value is very large. Thus, the gold antenna performs very similarly to a perfect electric conductor (PEC): the electric field inside antenna remains very small.

It has been found that when solving Maxwell's equations in frequency domain, anisotropic material such as calcite may lead to poor convergence if iterative solver is used. Therefore, the direct solver "PARDISO" is used in all our COMSOL simulations.

<u>Simulation details for Figure 1</u>

For the simulations shown in Figure 1, the simulation domain size in xy-plane is 75 μm $\times$ 50 μm. The frequency is set as $\omega = 1460\ cm^{-1}$. The gold antenna, with a thickness of 50 nm, is put at the center of xy-plane, just above the vacuum-calcite interface.

With the help of total-field scattered-field (TF-SF) technique, a p-polarized plane wave serves as the incident wave (incident angle set to be 60°). By putting a monitor at 100 nm above the calcite's surface, the near-field distribution can be recorded. Finally, the corresponding light intensity $|\vec{E}|^2$ is plotted, as shown in Figure 1d-f. Note that only the middle part of the simulation domain is shown in Figure 1, while artifacts close to the domain boundaries are cropped out. The corresponding Fourier transform (FT) results are obtained with the help of MATLAB R2021b's "fft2()" function.

Here we provide the shape parameters of the three gold antennas we've used:

(1) For the "disk" antenna, its radius is 0.75 μm.

(2) For the “rectangle” antenna, its length is 3.2 μm, while its width is 1.4 μm. Instead of using a perfect rectangular box, in COMSOL we construct this antenna using one rectangular box and two cylinders (with radius 0.7 μm). Therefore, we can keep the antenna shape used in simulation consistent with the one used in experiment.

(3) For the “triangle” antenna, its length is 8.4 μm, while its width is 4.9 μm. Based on similar reason, in COMSOL this antenna consists of three cylinders, three rectangular boxes, as well as one prism.

<u>Simulation details for Figure 3</u>

For the simulations shown in Figure 3, the simulation domain size in xy-plane is 75 μm ×50 μm. The frequency is set as $\omega = 1460\ cm^{-1}$. Note that here to show the directionality of emitted polariton, only the “triangle” antenna is used.

Similar to Figure 1, only the “triangle” antenna is used. The only difference is that here we try to rotate the antenna.

In order to demonstrate that the directionality of emitted polariton wave depends on the rotation angle of gold antenna, the light intensity distributions for multiple rotation angles $(\sigma = 0^{\circ}, 30^{\circ}, 60^{\circ}, 90^{\circ})$ are plotted.

Other parameters remain the same compared with Figure 1.

<u>Simulation details for Figure S6</u>

For the simulations shown in Figure S6, the simulation domain size in xy-plane is 75 μm ×50 μm. Similarly, a gold antenna, with a thickness of 50 nm, is put above the vacuum-calcite interface. Similar to Figure 1, only the “triangle” antenna is used. The only difference is that here we try to just change the excitation frequency.

.

In order to demonstrate that the directionality of emitted polariton wave strongly depends on the frequency, the light intensity distributions for multiple different frequencies ($\omega = 1430\ cm^{-1}$, $1450\ cm^{-1}$, $1470\ cm^{-1}$) are plotted.

Parameters regarding the incident wave, as well as other hyper-parameters, remain the same compared with Figure 1.

Supporting Information Note 3: Selective mode excitation of different shape nano antennas: directionality measurements and excited mode identification.

There are four polaritonic branches that are being studied in this work. The four branches have been defined based on the direction of polariton mode's group velocity. More specifically, we define the "U" branch and "L" branch based on the y component of group velocity (energy going to +y direction gives U, while -y gives L). On the other hand, the "+" and "-" are defined based on the x component of group velocity (energy going to +x direction gives +, while -x gives -). Combining these two would give 4 branches, which we call U+, U-, L+, L-. These 4 branches correspond to the 4 quadrants of the reciprocal space:

$$\text{U+: } k_x > 0,\, k_y < 0;\ \text{U-: } k_x < 0,\, k_y < 0.$$

$$\text{L+: } k_x > 0,\, k_y > 0;\ \text{L-: } k_x < 0,\, k_y > 0.$$

The two different angle parameters shown in Figure S2b and c are the angles of the incident wave φ (w.r.t the z axis) and ∅ (within x-y plane) that can be used to create an asymmetric distribution in polaritonic branches. This has been investigated recently by a few studies where the broken of symmetry for g-HPs[3], hyperbolic PhPs[4] and even bloch modes[5]. These techniques do have the capability to get broken symmetry but are limited in controlling the directionality of the polariton. Even when the incident angle of the excitation wave changes the formation of the asymmetric polaritonic rays depends on the weak and strong local fields generated on the left and right side of the nano antenna[1]. Therefore, the left and the right (+) polaritonic branches are no longer symmetric. The strong local fields (larger local photonic density of state) on the (+) polaritonic branch results in the superposition of many polaritonic modes with different wavevectors and thus showcases a ray like propagation. Figure S2d-f represents the symmetric and asymmetric distribution of the polaritons due to the change of incident angle. It is clear that the normal incident (Figure S2d) showcases a symmetric polariton distribution with similar propagations in all four polaritonic branches and this can be clearly visualized in the momentum space as illustrated in Figure S2g. There are four bright regions in the momentum space representing the four k vectors ($k_{L-}, k_{L+}, k_{U-}, k_{U+}$)) with an equal magnitude that justifies the symmetric distribution in the polariton wavefronts.

When φ changes from 90° to 60° as illustrated in Figure S2e the U- and L+ branches are much more intense and this intensity of those two branches increases if the incident angle is further reduced as evident in Figure S2f (where $\varphi = 30^{\circ}$). The corresponding FFT results (Figure S2h,i) confirm the asymmetry in the propagation through the brightness of the modes excited for each branch. Apart from this the asymmetry of the polariton can be achieved through changing the polarization angle ∅ (Figure S2c) as recently demonstrated in this

study[3]. Even in this study the symmetry is broken in a way that is achieved through changing of φ but with that only the intensity of the U- and the L+ branches can be controlled.

Here in our research, we have suppressed the lower branch completely by just changing the shape and the size of the micro/nano antenna and to demonstrate this we used two different techniques to evaluate the directionality of the polariton as explained in the main text. First the directionality of the generated g-HP is determined by comparing the polariton intensities of the upper and lower branches. The directionality constant β is defined as in Equation 1 in the main text. For the polaritons generated by the three shapes we calculated the amplitude intensity of the upper and lower branches of the polariton by considering a line cut profile for the obtained results (Figure S3a, d and g). The line cuts were produced such that the polariton intensity contrast (ratio between the highest and the lowest values) is the maximum. For the three main nano antenna shapes the directionality constant results are as follows (amplitude values shown in Figure S3c, f and i).

$$\beta_{disk} = \left|\frac{0.113}{0.108}\right| = 1.046$$

$$\beta_{rectangle} = \left|\frac{0.446}{0.149}\right| = 2.99$$

$$\beta_{triangle} = \left|\frac{0.474}{0.109}\right| = 4.348$$

Therefore, we can clearly see that the directionality can be modulated by changing the shape of the antenna.

$$\beta_{triangle} > \beta_{rectangle} > \beta_{disk} \qquad \text{(S11)}$$

Similarly for different orientations of the triangle (Figure S4) w.r.t the calcite surface we can obtain different directionality constant values, confirming that the directionality can be modified just by changing the orientation of the nano antenna. The following β values are calculated based on the amplitude intensities of the upper and lower polariton line cuts of the different orientations of the triangular nano antenna $\sigma = 0^\circ, 30^\circ, 60^\circ, 90^\circ$ (Figure S4e-h).

$$\beta_0 = \left|\frac{0.474}{0.109}\right| = 4.348$$

$$\beta_{30} = \left|\frac{0.251}{0.062}\right| = 4.04$$

$$\beta_{60} = \left|\frac{0.427}{0.233}\right| = 1.83$$

$$\beta_{90} = \left|\frac{0.308}{0.179}\right| = 1.72$$

$$\beta_0 > \beta_{30} > \beta_{60} > \beta_{90} \qquad \text{(S13)}$$

Secondly, simulation and experimental results were subjected to Fourier transform to reveal two distinct hyperbolic iso-frequency contours (IFCs) in k-space as shown in Figure S5. All the simulation results are $|E_z|$ field distribution on xy plane, while all the experimental results shown are amplitude signal S_2 obtained through s-SNOM. The amplitude (A) and the phase ($\emptyset$) of the polariton can be extracted from the SNOM second harmonic amplitude (O2A; Figure S7a) and phase (O2P; Figure S7b) images. This Amplitude and phase data is used to calculate the E field which is then used to produce the FT results as illustrated in Figure S7c.

$$E = A \times e^{i\emptyset} \tag{S12}$$

There are either single or multiple modes excited by the antenna depending on various parameters as evident by the FFT results (Figure S5). The k vectors and the group velocities drawn in main text Figure 1 were identified as illustrated in Figure S3j-o. Panels J to L represent the same set of FT results shown in main text Figure 1, but we have shown how the FFT amplitude intensities were measured using a line cut profile to determine the different modes excited by the differently shaped antennas. The line cut profiles (Figure S3m-o) showcase the excitation efficiency of the polariton modes by each shaped antenna. The dot and the rectangle are exciting some low k modes with a higher efficiency, but it is gradually decreasing showcasing low efficiencies in high k mode excitation. One of the two modes shown in Figure 1g (represented with wave vector K_{L-}) is the M_{1D} mode. This is the largest k vector with a reasonable coupling efficiency that a 1.5 μm disk can excite. A smaller disk can excite much larger k vectors. The 1.5 μm roughly produces all the low k vectors in between K_{L-} and K_{u-}. Similarly, the K_{u-} mode shown in Figure 1h corresponds to the M_{1R} mode which is the mode that the rectangle antenna excites with the highest efficiency. But the FT for the triangle (Figure S3l) and the line cut profile (Figure S3o) clearly show that the triangle antenna can excite multiple discrete modes with different efficiencies. The K_{u-} wave vector in Figure 1i is drawn to represent one of these highly efficient modes (M_{2T}) that a triangle antenna excites.

The comparison of the intensities of each branch through β and the polaritonic mode analysis through the FT results were crucial to properly identifying the selective mode excitation that is achieved through engineering the micro/nano antenna. Even for the rotated triangles the excited modes are clearly visible through the FFT results shown in Figure S4i-l. The FFT results for Figure 1 from both experimental and simulation data is illustrated in Figure S5. These IFCs correspond to the left and right parts of the polariton generated by each shape, with the outer and inner IFCs representing the left and right polaritons, respectively. It can be

observed that the left polariton exhibits a concaved wavefront, whereas the right polariton wave is diffractionless.

We intended to design the micro/nano antenna to achieve more directionality and long propagation. First, we performed a series of simulations (see Figure S8) to determine the effect of different diameters of a disk antenna. To confirm, we investigated polariton excitation behavior through the experimental near field results for a few different disk antennas with increasing diameters (see Figure S9a-c). When the diameter increases the electric field distribution gets complex allowing multiple curved points in the antenna edge to allow exciting polaritons. As illustrated in Figure S9c the disk antenna (radius = 2 μm) showcases four curved points (A-D) which polariton rays are excited. When the diameter decreases these points come closer to each other resulting in highly confined polariton excitations. If the disk structure is extended in the y or x axis to generate an ellipse shape, we observed that two curved points dominate in exciting polariton modes. As illustrated in Figure S9d the vertical ellipse with a major axis in the y direction (major axis = 8 μm, minor axis = 4 μm) excites two portions at curved points C and D. The interference fringes of both those excited polaritons (at points C and D) towards the left side (- branch) can be observed clearly. Then we extended the design to a triangle which consists of three curved points (vertices of the triangle) named A, B and C (see Figure S9i left panel; nearfield image). These three vertices will act as the polariton excitation points, and the curvature will determine the most efficient modes excited by the curved points of the triangle micro antenna.

For a triangle shaped antenna, the left side polariton branches (- branch) are assisted by the collective excitation by the two vertices A and C of the triangle. By changing the curvature of the curved point A and C, the excited mode can be altered. Then the inclined edge AC of the triangle can either enhance or suppress the excited polariton modes. This is explained in Supporting information note 4. Therefore, the selective excitation of a triangle is determined by the curvature of the three vertices and the edge orientation of the triangular shape.

Supporting Information Note 4: Edge assisted mode selection.

The rectangular and triangular-shaped micro antennas employed here for polariton excitation feature notably larger physical edges. These edges can direct reflected light towards the same direction that is perpendicular to the specific physical edge of the nano antenna under specific conditions[6]. At the same time the edge assists to enhance or suppress the excited polariton modes if specific modes are excited by the antenna. This section provides insights into the design parameters governing these physical edges for the purpose of controlling edge assisted mode selection. The anisotropy of the calcite material, which is a hyperbolic material, can be effectively characterized through its IFC, represented in the following manner:

$$\frac{x^2}{c_x} - \frac{y^2}{c_y} = 1 \tag{S14}$$

Where, $c_x = Re(\varepsilon_{\parallel})$ and $c_y = Re(\varepsilon_{\parallel} \sin^2\theta + \varepsilon_{\perp} \cos^2\theta)$

As illustrated in Figure 2f $\boldsymbol{k_i}$ represents the incident wave in the momentum space and the $\boldsymbol{k_i}$ vector can be written as:

$$\boldsymbol{k_i} = \hat{x}k_{ix} + \hat{y}k_{iy} \tag{S15}$$

Where$k_{ix} = k_0 \cos\varphi \times \cos\emptyset$ and $k_{iy} = k_0 \cos\varphi \times \sin\emptyset$.

In our work the tip generated polariton is not reflected by the physical edge of the antenna. The tip generated polariton does not propagate long distances in calcite since the tip is extremely small. As illustrated Figure 2f schematic if the vertex of the triangle antenna (A) excite a mode which has a k vector perpendicular to the AC physical edge the edge supports those modes and enhances the excitation efficiency to allow a single mode to propagate. Interestingly the curvature of the vertex C can be adjusted to allow polariton modes in the U-branch which has wave vectors perpendicular to the edge and then the edge can enhance its coupling efficiency. Therefore, the A curved vertex, AC physical edge and the C vertex collectively couples the energy of the incident light to a single polariton mode allowing parallel fringes to the physical edge.

We can get the relationship between the internal angle of the triangle δ and the selectively excited k vector $\boldsymbol{k_e}$ through the following:

$$\delta = 90^{\circ} - \tan^{-1}\left(\sqrt{\frac{c_y}{c_x} \times \frac{(k_e^2 - c_x)}{(k_e^2 + c_y)}}\right) \tag{S16}$$

According to Equation S16, for a specific excitation frequency below a critical δ angle the edge cannot support any edge assisted modes. To verify this, we simulated two distinct triangles, representing the green and blue colored triangles from Figure 2f (with internal angles δ_1 and δ_3). The simulations were performed with an illumination frequency of $\omega =$

$1460\ cm^{-1}$. The simulation outcome for the larger triangle ($\delta_3 = 70^{\circ}$) is illustrated in Figure S10a. The FT result for this polariton excitation (Figure S10c) shows one bright spot at the coincident point of the IFC and line l_{t1}. This perfectly validates the edge assisted mode selection that can be achieved by varying the angle of the AC physical edge. In contrast, the simulated a triangle with $\delta_1 = 30^{\circ}$ (Figure S10b) does not show parallel fringes to the AC physical edge, and the corresponding FT result reveals multiple excited modes. However, there is no intersection point between the IFC and line l_{t3} confirming the absence of edge-supported mode selection (Figure S10d).

Supporting Information Note 5: Propagation length of different g-HP modes.

In this section, we introduce details regarding how the propagation length of g-HP modes can be predicted theoretically[4]. By numerically calculating the mode profiles and propagation lengths of all g-HP modes on the IFC, we believe that this note provides important details related to the major theoretical findings of this paper.

In general, in order to find the dispersion relation of g-HP modes numerically, for a given (k_x, k_y) we first need to figure out the $\vec{E}, \vec{H}$ field distribution of eigenmodes that exist inside air or calcite substrate. By expressing the g-HP surface mode as a superposition of these eigenmodes, and imposing the electromagnetic boundary conditions at the $z = 0$ interface, it'd be straightforward to calculate the field distribution, as well as the propagation length, of an arbitrary g-HP mode.To remain consistent with S1, we assume plane wave solutions with $e^{i\vec{k}\cdot\vec{r}}$ dependence. The g-HP mode is a combination of eigenmodes inside air and eigenmodes inside calcite. Denote the wave vector of plane wave solutions inside air as $\vec{k}_1 = [k_x, k_y, k_{1z}]$, where $k_{1z} = i\sqrt{k_x^2 + k_y^2 - k_0^2}$. The eigenmodes can be decomposed into two independent solutions, correspond to the plane wave solutions with TM and TE polarizations:

$$\text{TM: } \vec{E}^{\text{TM}} \sim [-k_x k_{1z}, -k_y k_{1z}, k_x^2 + k_y^2];$$

$$\text{TE: } \vec{E}^{\text{TE}} \sim [-k_y, k_x, 0].$$

For eigenmodes inside calcite, denote the wave vector as $\vec{k}_2 = [k_x, k_y, k_{2z}]$. Again, two solutions exist: for the ordinary wave (o-wave), we use $k_{2z} = -i\sqrt{k_x^2 + k_y^2 - \epsilon_\perp k_0^2}$ to make sure that the electric field decays to zero when $z \to -\infty$; for the extraordinary wave (e-wave), two k_{2z} solutions can be found in general by solving quadratic equation, as stated Supporting Information note 1. Note that only the solution which satisfies $\text{Im}[k_{2z}] < 0$ will be used Once the valid k_{2z} values have been determined, the corresponding electric field $\vec{E}^o$ (o-wave) and $\vec{E}^e$ (e-wave) can be derived by solving the source-free Maxwell's equation:

$$\vec{k}_2 \times \vec{k}_2 \times \vec{E} + k_0^2 \bar{\bar{\epsilon}} \cdot \vec{E} = 0. \tag{S17}$$

which is a linear equation w.r.t. E_x, E_y, E_z components. In order to derive the dispersion relationship of g-HP modes, we now focus on a linear combination of the above 4 modes and impose the continuous conditions for tangential field components E_x, E_y, H_x, H_y at $z = 0$ interface. More specifically, in $z > 0$ region the electric field distribution can be writte as $\vec{E}(z > 0) = a^{\text{TE}}\vec{E}^{\text{TE}} + a^{\text{TM}}\vec{E}^{\text{TM}}$, while in $z < 0$ region the electric field distribution can be

writte as $\vec{E}(z<0)=a^o\vec{E}^o+a^e\vec{E}^e$, where $a^{\mathrm{TM}}/a^{\mathrm{TE}}$ and a^o/a^e are the coefficients of eigenmodes inside air and calcite, respectively. The corresponding 4 linear equations can be derived as.

$$\begin{pmatrix} E_x^o & E_x^e & -E_x^{\mathrm{TM}} & -E_x^{\mathrm{TE}} \\ E_y^o & E_y^e & -E_y^{\mathrm{TM}} & -E_y^{\mathrm{TE}} \\ H_x^o & H_x^e & -H_x^{\mathrm{TM}} & -H_x^{\mathrm{TE}} \\ H_y^o & H_y^e & -H_y^{\mathrm{TM}} & -H_y^{\mathrm{TE}} \end{pmatrix}\begin{pmatrix} a^o \\ a^e \\ a^{\mathrm{TM}} \\ a^{\mathrm{TE}} \end{pmatrix}=\vec{0} \tag{S18}$$

The fact that this equation has non-zero solution for $\vec{a}$ indicates that the following determinant equals zero:

$$\det(A)=\begin{vmatrix} E_x^o & E_x^e & -E_x^{\mathrm{TM}} & -E_x^{\mathrm{TE}} \\ E_y^o & E_y^e & -E_y^{\mathrm{TM}} & -E_y^{\mathrm{TE}} \\ H_x^o & H_x^e & -H_x^{\mathrm{TM}} & -H_x^{\mathrm{TE}} \\ H_y^o & H_y^e & -H_y^{\mathrm{TM}} & -H_y^{\mathrm{TE}} \end{vmatrix}=0 \tag{S19}$$

In order to find the field distribution of g-HP modes, the above equation needs to be solved. More specifically, we first set the in-plane wave vector (k_x, k_y) using a initialization point $(k_x^{\mathrm{init}}, k_y^{\mathrm{init}})$ that's close to the IFC. Then we solve the following optimization problem:

$$\min_{(k_x,k_y)} |\det(A)|^2,$$

in which the relationship between matrix A and the in-plane wave vector (k_x, k_y) has already been defined. Theis optimization problem is solved with the help of fmincon() function provided by MATLAB R2021b. In the end, for any given g-HP mode that's lying on the IFC, both the real and imaginary part of k_x, k_y can be calculated numerically. . The corresponding coefficients a^{TM}, a^{TE}, a^o, a^e, as well as the electromagnetic field distributions, can thus be obtained numerically.

For a certain g-HP mode obtained from the above procedure, we now focus on its propagation length L_p, defined as the length at which the electric field's magnitude decays to $1/e$. The propagation length is related to the imaginary part of the in-plane wave vector, and can be estimated numerically using the following equation:

$$L_p=\frac{1}{\sqrt{\mathrm{Im}(k_x)^2+\mathrm{Im}(k_y)^2}} \tag{S20}$$

In order to understand the existence of long-propagating g-HP modes better, here we visualize the simulated propagation lengths (normalized by wavelength λ_0 inside vacuum) of all g-HP modes on the IFC in Figure S11. There are several points that worth paying attention to:

(1) While high-k g-HP modes cannot propagate long, the propagation length L_p can be pretty large ($> 10\lambda_0$) for certain low-k g-HP modes. For example, at 1470 cm^{-1} the predicted L_p of the g-HP mode located at $(\text{Re}k_x, \text{Re}k_y) = (-1.544k_0, -0.295k_0)$ can exceed 162.7 μm, which is remarkable and justifies the improvement of our work.

(2) For small $|k_y|$, no valid g-HP solution can be found numerically, meaning that g-HP mode does not exist if the angle is too small. We've verified that this behavior comes from the fact that neither of the two k_{2z} solutions for e-wave can satisfy the $\text{Im}(k_{2z}) < 0$ condition. Without a valid e-wave mode, the g-HP mode cannot be constructed by imposing the boundary conditions, leading to a large $|\det(A)|$ value.

(3) The directionality angle increases when the frequency increases, which is consistent to literatures as well as our experimental results.

We would like to mention that at 1460 cm^{-1} the predicted propagation length L_p can be larger than 119.5 μm for the g-HP mode located at $(\text{Re}k_x, \text{Re}k_y) = (-1.590k_0, 0.588k_0)$. This is consistent with the fact that we have observed a long-propagating g-HP mode ($L >$ 82.3 μm) at 1460 cm^{-1} in our experiment.

Supporting Information Note 6: Relationship between multi-mode excitation and short propagation length

In Supporting Information Note 5, we have already proved numerically the existence of long-propagating g-HP modes on calcite surface. For a certain g-HP mode, once excited, its propagation length does not change. Considering the fact that a small disk antenna serves as a dipole source[3] and should be able to excite all g-HP modes (including both high-k modes that decay fast, and low-k modes that decay much slower), it may seem counter-intuitive that such long-propagating g-HPs have never been observed in literatures. In this note, we aim to address this question. We point out that while a disk antenna can excite both long-propagating modes and short-propagating modes simultaneously, the energy of the incident light gets distributed into multiple g-HP modes, making the long-propagating modes difficult to observe.

It has been pointed out that when a dipole source is placed inside a structured photonic environment[7], the emitted energy would get distributed among multiple different electromagnetic modes, leading to a short dipole-dipole interaction range. A similar analysis can be carried out in our system. As shown in main text Figure 4a, a single dipole pointing along the z-axis can excite multiple g-HP modes simultaneously. On the other hand, a dipole array containing $N = 51$ dipoles (interval between neighboring dipoles is $d = 0.05\lambda_0$) excites g-HP selectively. The E_z distribution along dotted lines are plotted in Figure 4b. Limiting the g-HP modes excited enhances the propagation length by approximately 4 times. Notice that the photonic environment (the calcite-air interface) does not change for the above two cases, thus the supported g-HP modes, as well as their propagation lengths, do not change. The above simulation results support our claim, showing that by simply changing the way of polariton excitation, the decaying pattern of the resulting $\vec{E}$ field distribution can differ substantially. By matching the orientation of dipole array with the wavefront of a long-propagating g-HP mode, the excited polariton wave can be both long-propagating and highly-directional.

In reality, it would be difficult to construct an array of ideal dipole sources. In this work, we achieve highly-selective g-HP mode excitation by using the edge of a triangular gold antenna. Both s-SNOM measurements and FFT results have shown that this edge-assisted excitation strategy is pretty effective. By coupling the incident plane wave strongly to a certain mode (whose wavefront is determined by the edge orientation), we have successfully excited the predicted long-propagating g-HP mode. The line-cut profile data taken from the four s-SNOM

images in main text Figure 4d,f and Figure S11e,g are fitted using a decaying sinusoidal function, as given in Equation S21:

$$y(x) = y_0 + A \times e^{-dx} \times \sin\left(\frac{\pi(x-x_c)}{w}\right) \tag{S21}$$

where y_0 = offset, A = amplitude, d = decay constant, x_c = phase shift, w = period.The propagation length obtained from curve-fitting can go beyond 82 μm. As a comparison, in previous works the g-HP excited by a disk antenna cannot propagate longer than ~ 20 μm. This result remains consistent with our previous explanation, showing that although a disk antenna can excite g-HP modes that propagate in different directions, most of these modes decay fast. This leads to a restricted propagation length and hinders the observation of long-propagating modes.

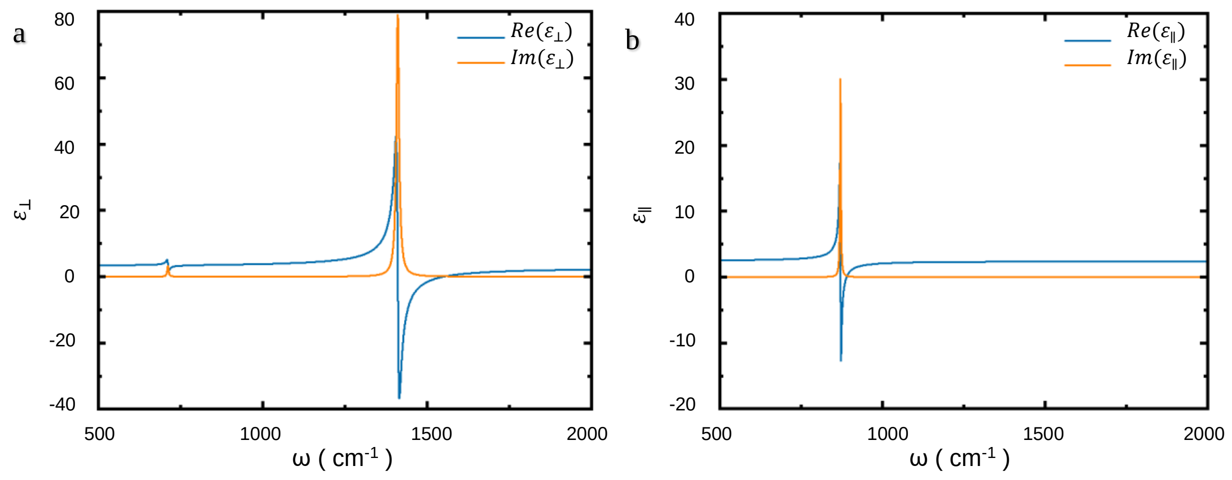

Figure S1. Calcite's permittivity calculated using the Lorentz oscillator model. (**a**) Real and imaginary parts of calcite's permittivity tensor component $\epsilon_\perp$. (**b**) Real and imaginary parts of calcite's permittivity tensor component $\epsilon_\parallel$.

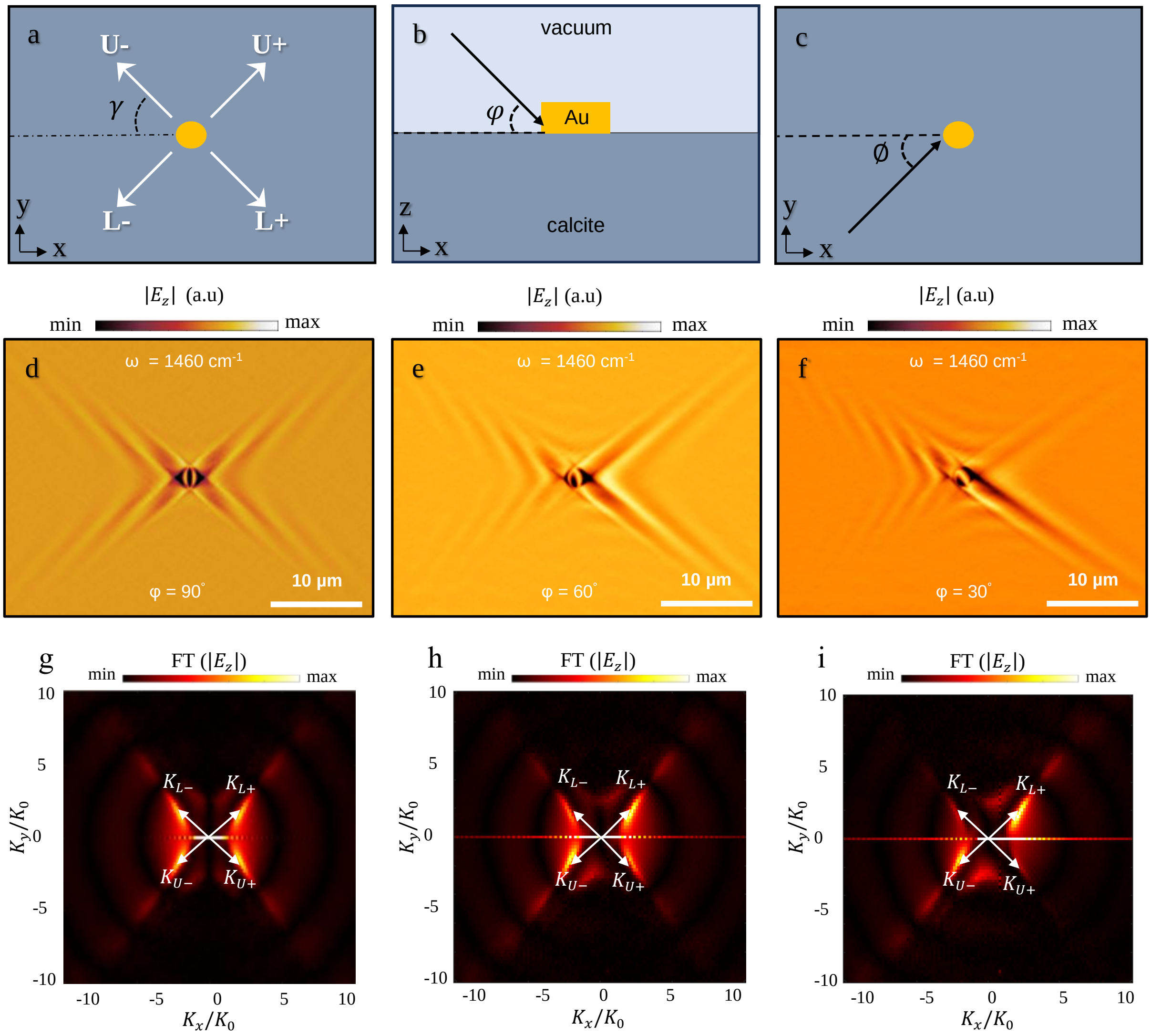

Figure S2. Polariton parameters and introducing asymmetric excitation. (**a**) Schematic illustration of the four branches of polaritons U+ (upper positive), U- (upper negative), L+ (lower positive), L- (lower negative) and the propagation direction angle γ. (**b**) Schematic illustration of the incident angle w.r.t z axis $\boldsymbol{\varphi}$. (c) Schematic illustration of the polarization angle which is the incident angle of the photon within the x-y plane. (**d-f**), Simulated near-field images of antenna-launched g-HPs at the illumination frequency of $\boldsymbol{\omega = 1460\ cm^{-1}}$ with an incident angle of $\boldsymbol{\varphi} = \mathbf{90^{\circ}, 60^{\circ}, 30^{\circ}}$. (**g-i**), Fourier transformed $|\boldsymbol{E_z}|$, generated by the disk antennas in d-f. white arrows represent the wave vectors of the excited modes by the antenna.

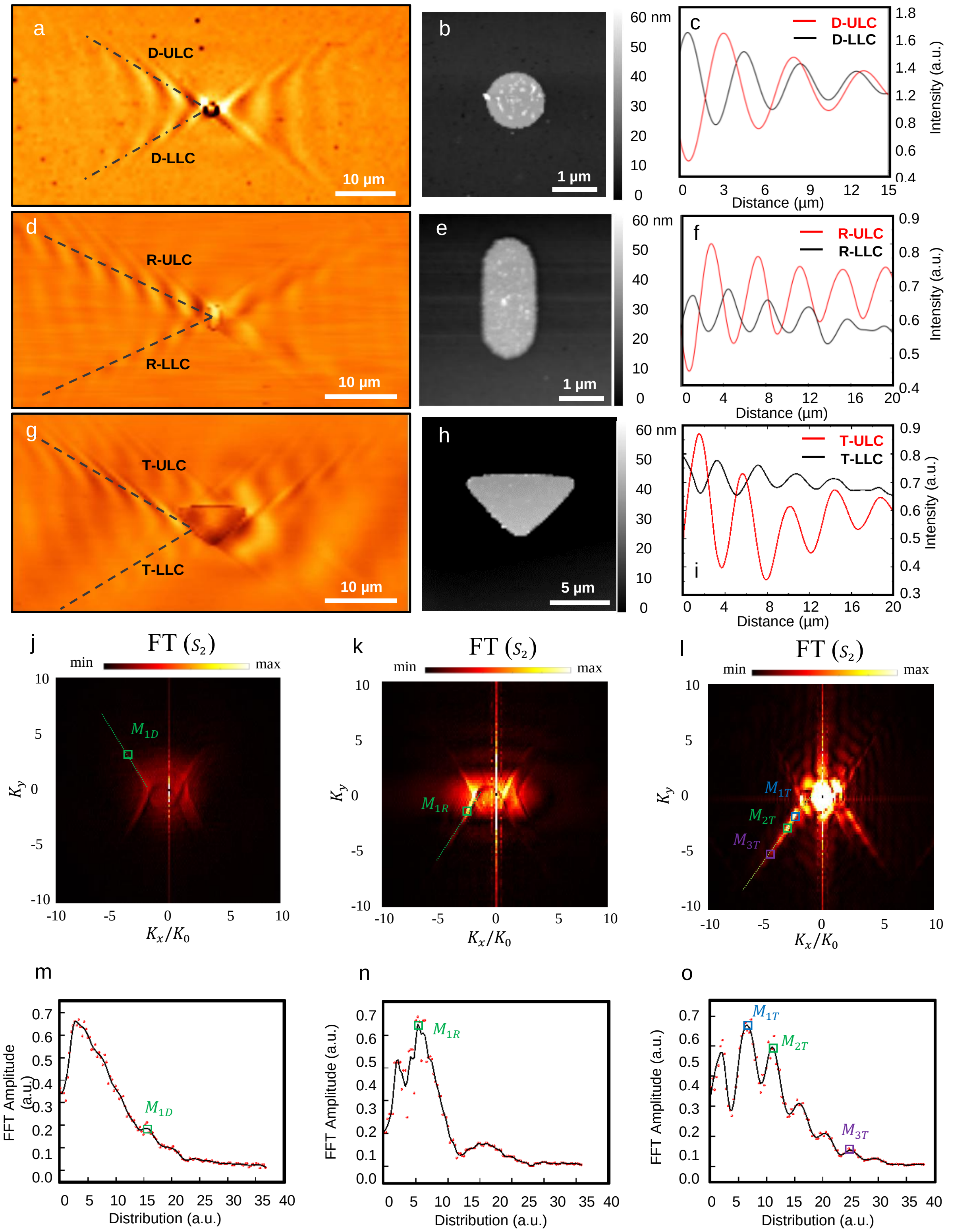

Figure S3. Determination of directionality of g-HPs for different shapes. (**a**) 50μm × 25μm s-SNOM scan of a disk-shaped antenna marked with the upper line cut (D-ULC) and lower line cut (D-LLC). (**b**) AFM image of the disk antenna (diameter = 1.5 μm). (**c**) Upper and lower line cut intensity profiles of the disk antenna shown in A. (**d**) 50μm × 25μm s-SNOM scan of a rectangle shaped antenna marked with the upper line cut (R-ULC) and lower

line cut (R-LLC). (**e**) AFM image of the rectangle antenna (W × H = 1.2 µm x 3.2 µm). (**f**) Upper and lower line cut intensity profiles of the rectangle antenna in D. (**g**) 50µm × 25µm s-SNOM scan of a triangle shaped antenna marked with the upper line cut (T-ULC) and lower line cut (T-LLC). (**h**) AFM image of the triangle antenna (L × H = 8.8 µm × 4.9 µm). (**i**) T-ULC and T-LLC intensity profiles of G. (**j-l**) Absolute value of the Fourier transform of the images shown in A, D, G. (**m-o**) FFT amplitude distribution along the line cut profiles shown in J, K and L. M_{1D} represents a mode excited by the disk antenna related to the peak shown in panel M. M_{1R} is the most prominent mode excited by the rectangular antenna corresponding to the peak shown in N. M_{1T} , M_{2T} , M_{3T} are 3 discreate modes excited by the triangle shape antenna with a high efficiency corresponding to the peaks shown in o. Green dotted line is used to produce the intensity line cut profiles shown in panels m-o.

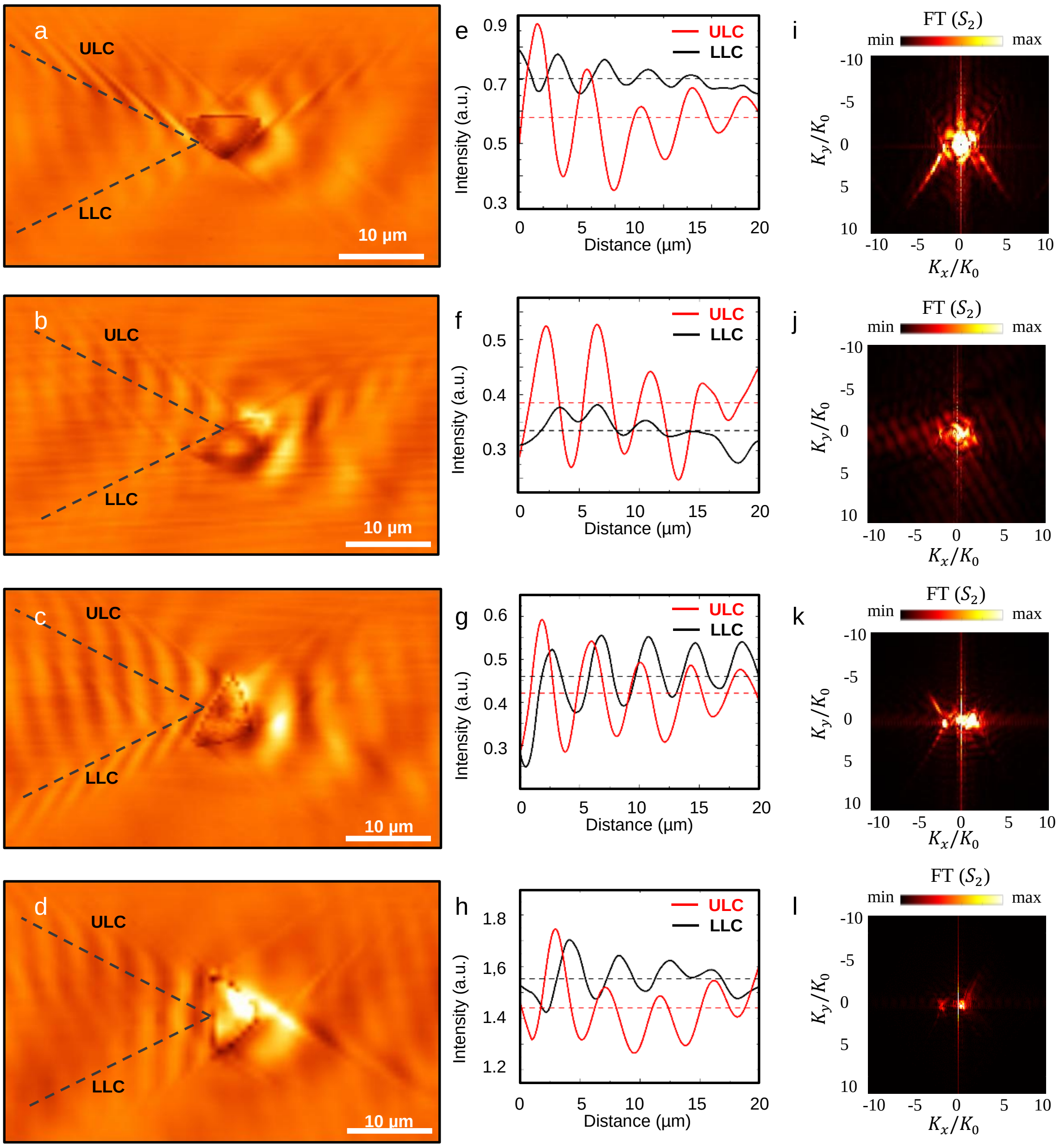

Figure S4 | Determination of directionality of g-HPs for different shapes. **(a-d)** 50µm × 30µm s-SNOM scan of a triangle-shaped nano antenna marked with the upper line cut (ULC) and lower line cut (LLC) profiles for the rotational angles β = 0°, 30°, 60°, 90°. **(e-h)** Upper and lower line cut intensity profiles of s-SNOM images presented in a-d. **(i-l)** Absolute value of the Fourier transform of the images shown in a-d.

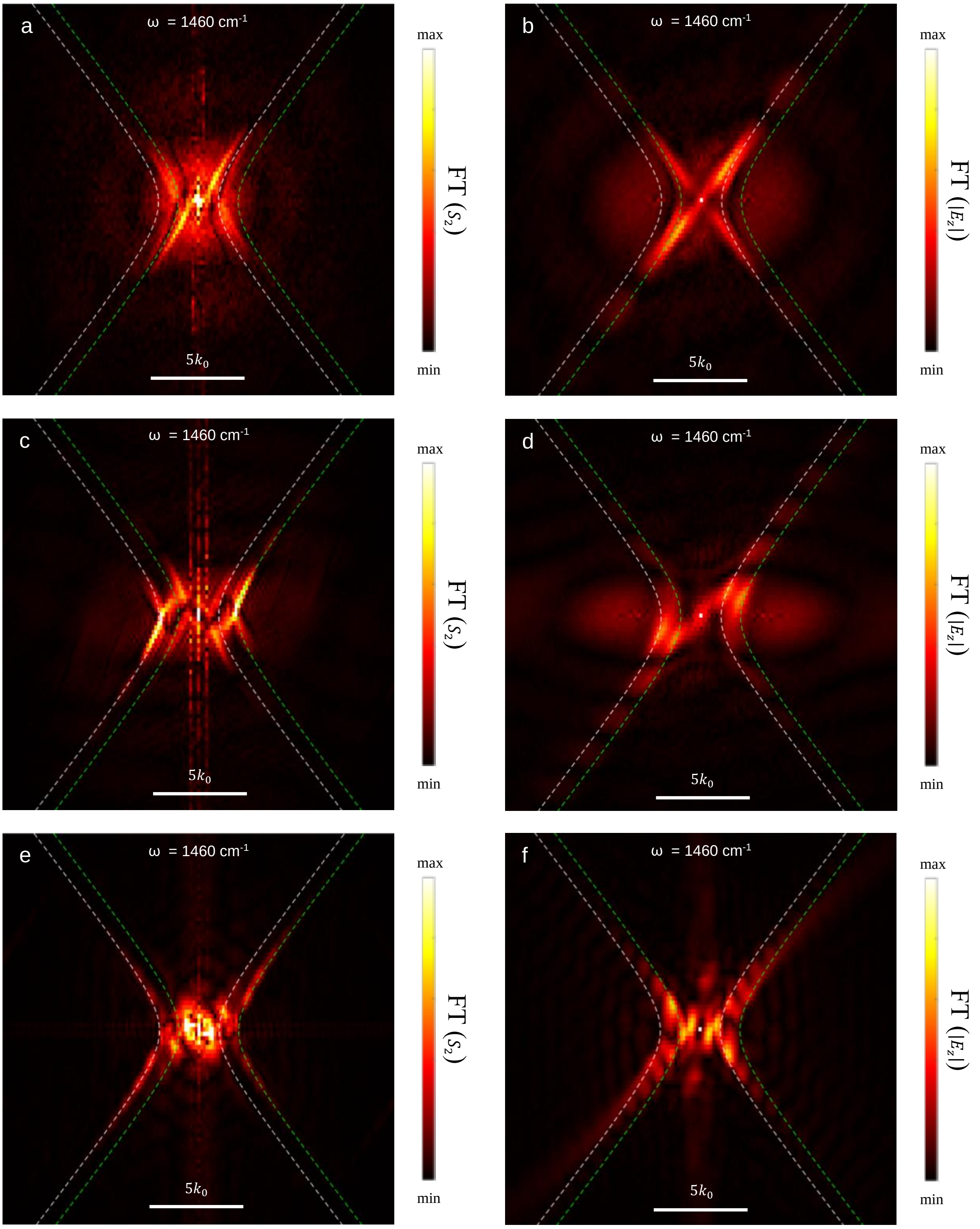

Figure S5 | Fourier transformation analysis of polaritons generated by different shaped nano antennas. (**a, b**) Fourier transformed S_2 and $|E_z|$, generated by the disk antenna shown in figure 1(A and D) in the main text. (**c, d**) Fourier transformed S_2 and $|E_z|$, generated by the rectangle antenna shown in figure 1(b and e). (**e, f**) Fourier transformed S_2 and $|E_z|$, generated by the triangle antenna showed in figure 1(c, f). The left column corresponds to experimental results, while the right column corresponds to simulation results.

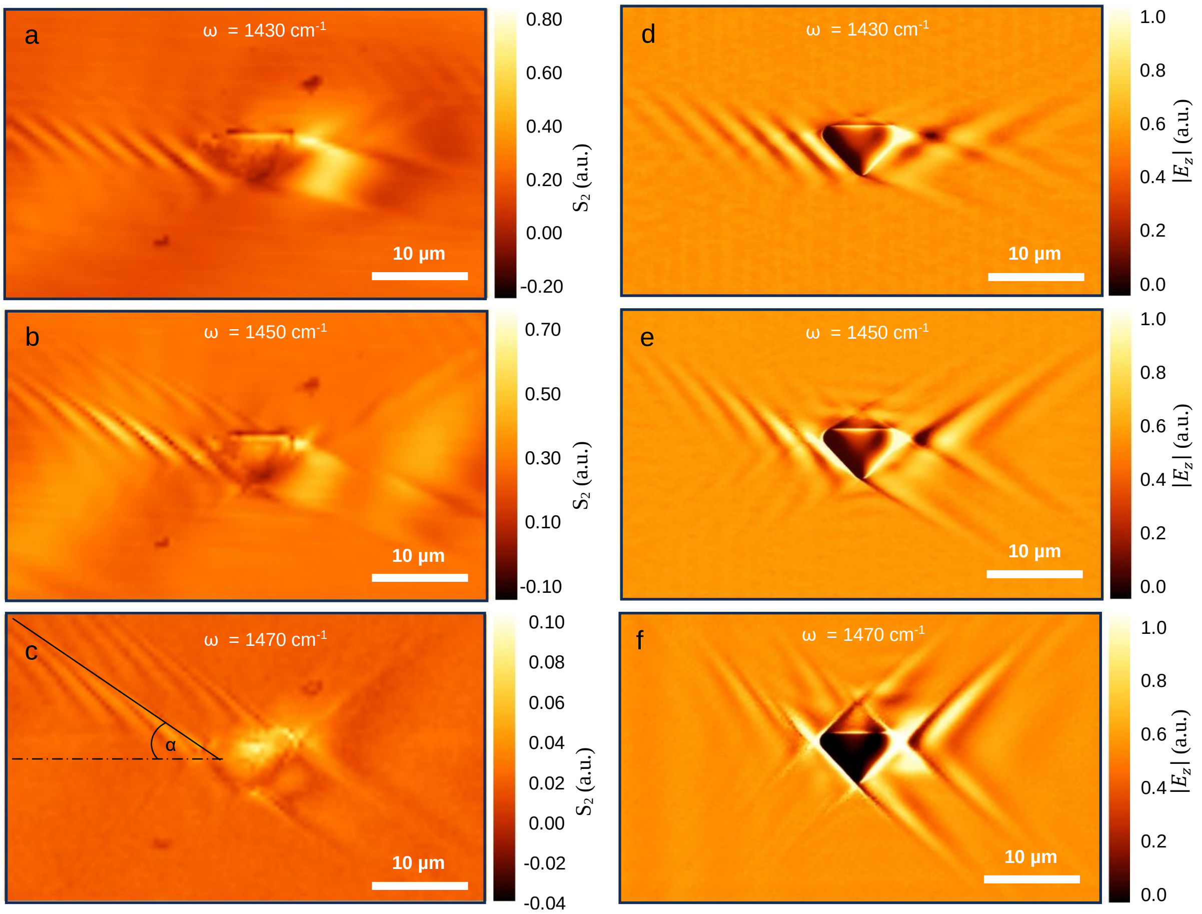

Figure S6 | g-HP excitation using a triangular antenna for different excitation frequencies. a-c, Experimental near-field images of antenna-launched g-HPs at the illumination frequencies of $\omega = 1430\ cm^{-1}$, $\omega = 1450\ cm^{-1}$, $\omega = 1470\ cm^{-1}$. α in panel c is the directionality angle of the excited polariton. **d-f,** Simulated near-field images of antenna-launched g-HPs at the illumination frequencies of $\omega = 1430\ cm^{-1}$, $\omega = 1450\ cm^{-1}$, $\omega = 1470\ cm^{-1}$.

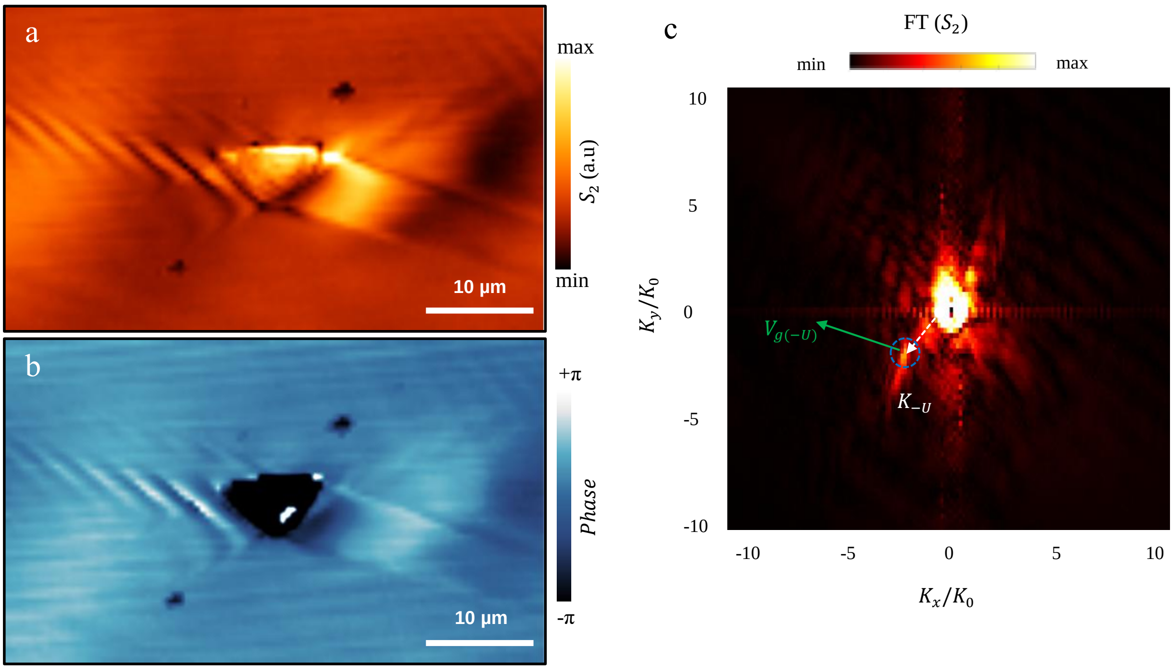

Figure S7 | Fourier Analysis calculation technique of the s-SNOM images. (**a**) Amplitude signal of experimental near-field images of a triangular shaped antenna-launched g-HPs at the illumination frequency $\omega = 1440\ cm^{-1}$. (**b**) Phase signal of experimental near-field images of a triangular shaped antenna-launched g-HPs at the illumination frequency $\omega = 1440\ cm^{-1}$. (**c**) Fourier transformed S_2, generated by the triangular antenna shown in a and b. dotted blue circle highlights the excited mode (bright spot), white arrow indicates the wave vector of the excited mode, and the green arrow indicates the group velocity of the polariton.

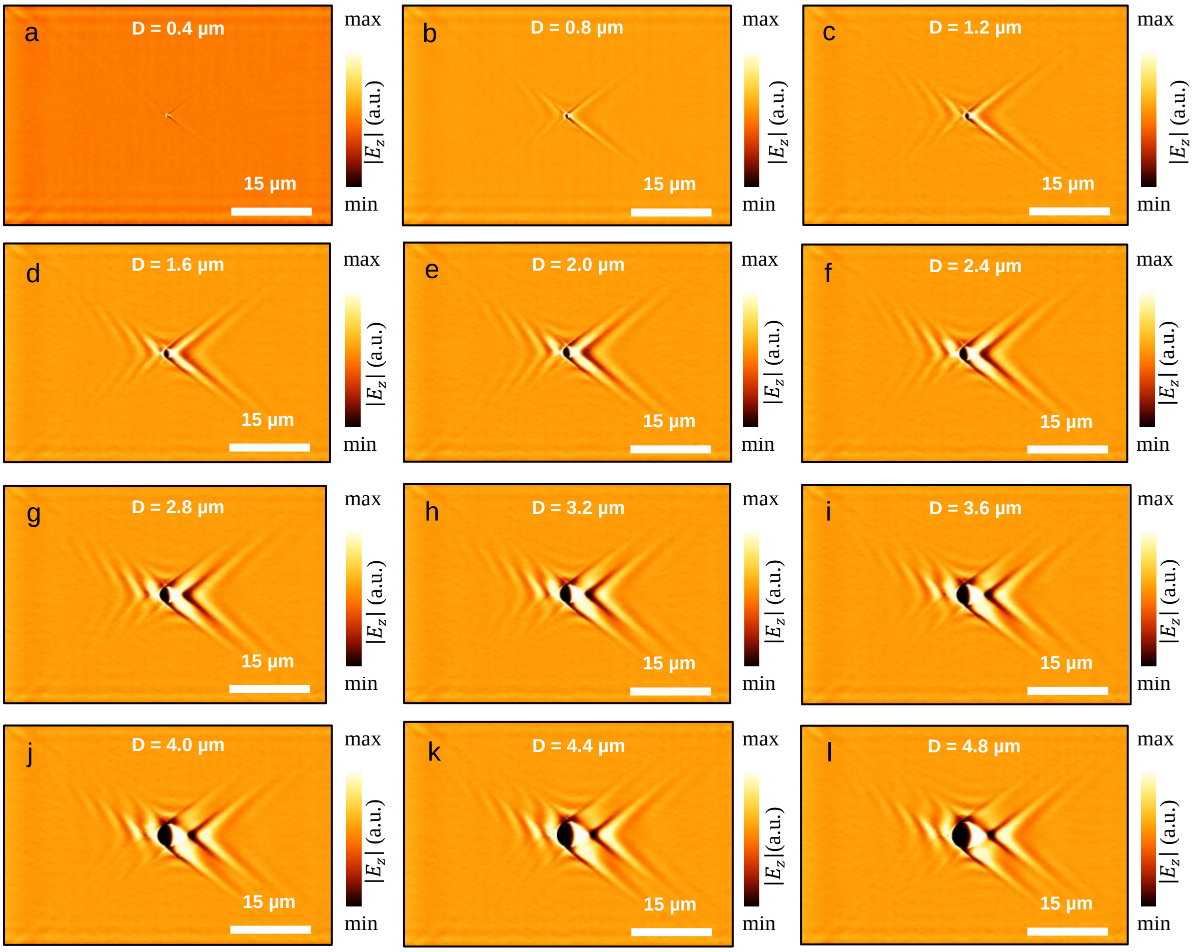

Figure S8 | Polariton propagation length comparison of different sized disk nano antennas. a-l, simulated near-field images of disk launched hyperbolic polaritons for different sized disk antennas for the illumination frequency 1460 cm^{-1}.

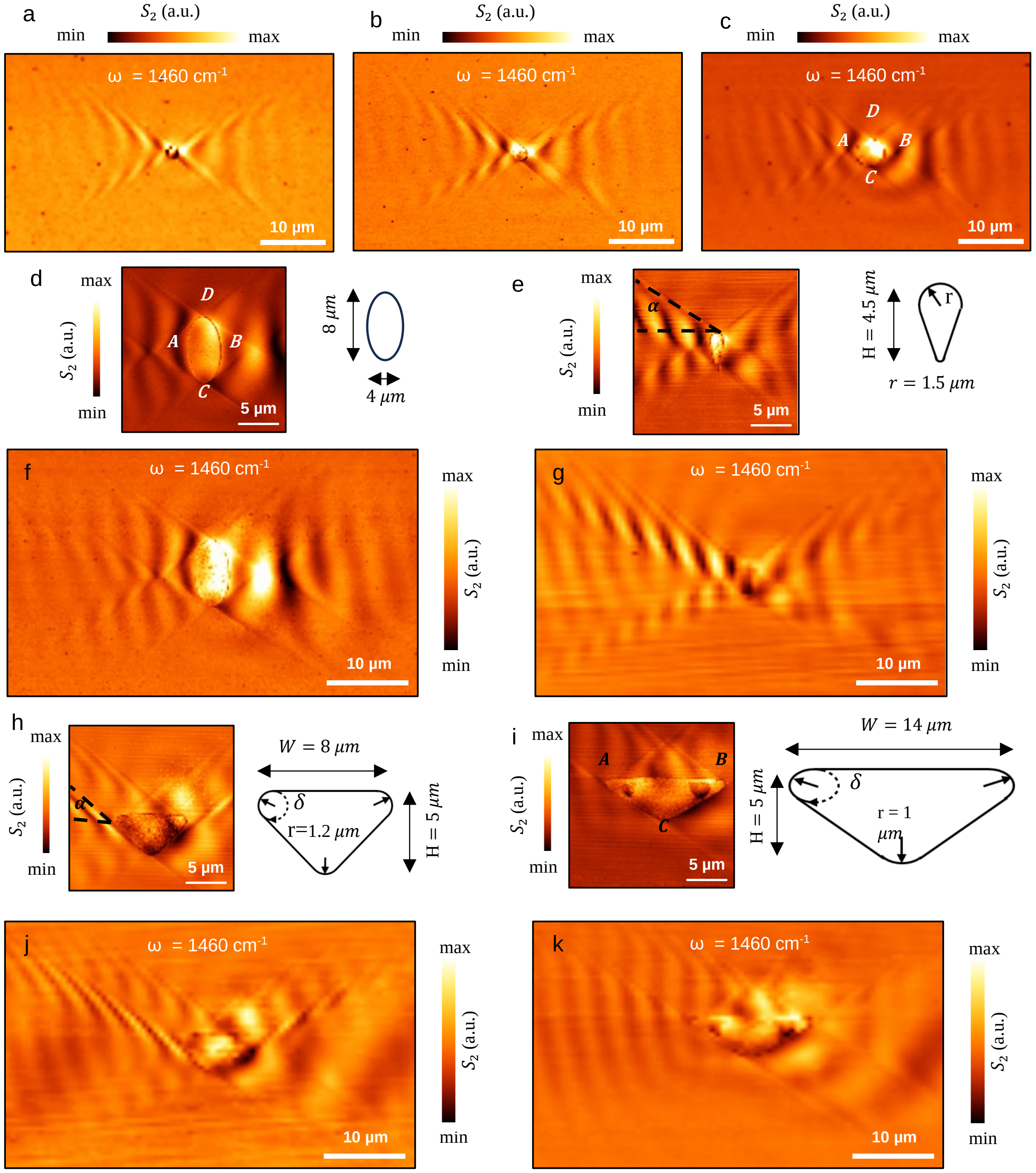

Figure S9 | Polariton excitation behavior from a disk antenna to a triangular antenna based on curvature and edges. (**a-c**) 50 × 30μm s-SNOM scan of the disk-shaped antennas with a radius of 0.75μm, 1 μm and 2 μm. A-D are the 4 curved points on the edge of the disk-shaped antenna. (**d**) 20 × 20μm near field image and the schematic diagram of an ellipse shaped antenna with a minor axis diameter of 4 μm and a major axis diameter of 8 μm. A-D are the 4 curved points on the edge of the ellipse-shaped antenna. (**e**) 20 × 20μm near field image and the schematic diagram of a small triangular shaped antenna. Curved point D with a radius of curvature 1.5 μm and a height of 4.5 μm. Open angle (α) indicated in between the

two dotted lines. (**f**) 50 × 30μm s-SNOM scan of the ellipse antenna shown in d. (**g**) 50 × 30μm s-SNOM scan of the triangle antenna shown in e. (**h**) 20 × 20μm near field image and the schematic diagram of a triangular shaped antenna. Curved points at A, B and C with a radius of curvatures 1.2 μm and a height of 5 μm. (**i**) 20 × 20μm near field image and the schematic diagram of a small triangular shaped antenna. Curved point at A, B and C with a radius of curvatures 1 μm and a height of 5 μm. Open angle (α) indicated by the two dotted lines. (**j**) 50 × 30μm s-SNOM scan of the triangle antenna shown in h. [1] 50 × 30μm s-SNOM scan of the triangle antenna shown in i. δ is the triangular internal angle which represents the inclination of the side of the triangle shape.

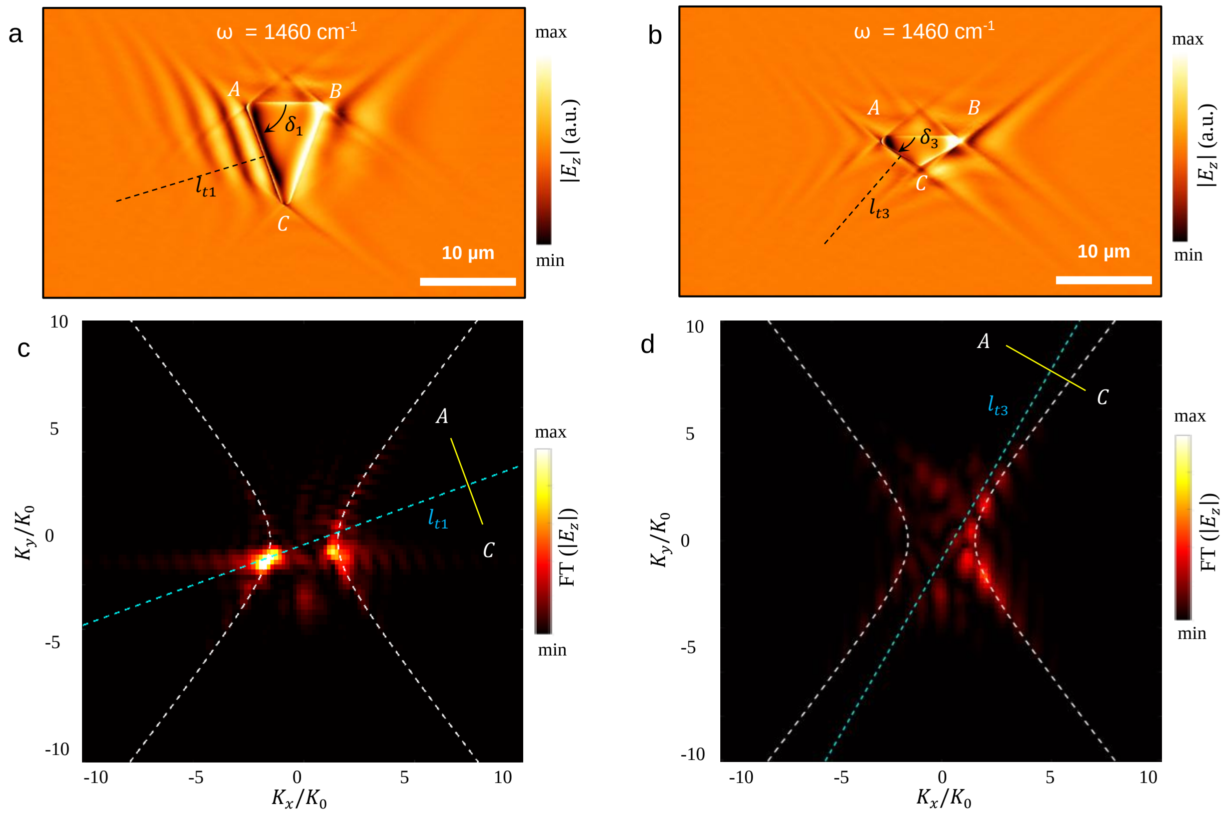

Figure S10 | Effect of negative reflection on directionality for rotated nano antennas. (**a, b**) Simulated near-field images of antenna-launched g-HPs for triangles with two distinct internal angles (δ). $\delta_1 = 70^{\circ}$ and $\delta_2 = 30^{\circ}$. (**c, d**) Absolute value of the Fourier transform of the images a and b. l_{t1} and l_{t2} are the perpendicular axis to the AC physical edge of the triangles with internal angles δ_1 and δ_2.

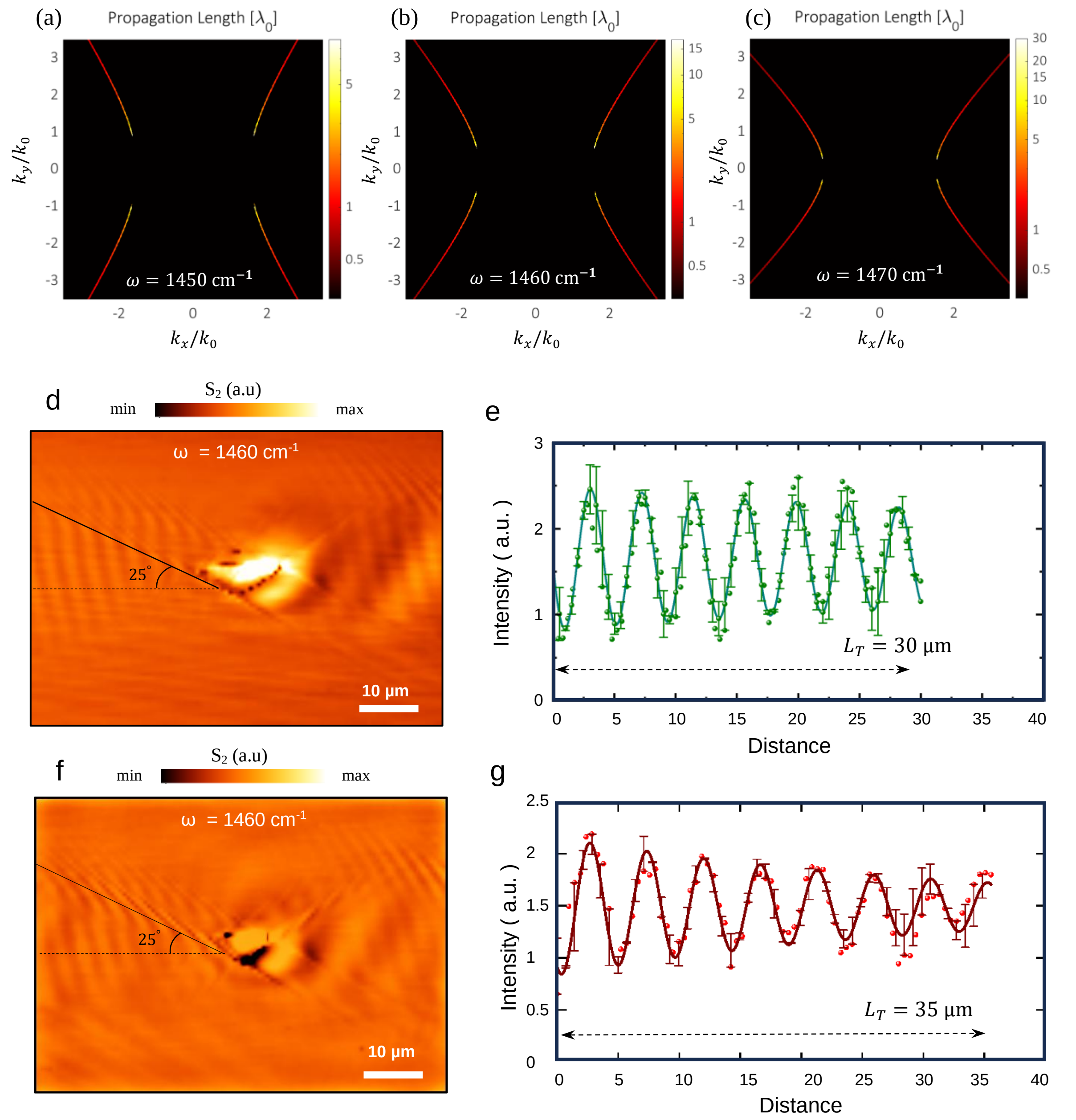

Figure S11 | Propagation lengths of g-HP modes on the IFC, visualized in momentum space. (**a - c**) Numerically calculated propagation lengths normalized using the wavelength λ_0 inside vacuum for excitation at $\omega = 1450\ cm^{-1}, 1460\ cm^{-1}\ and\ 1470\ cm^{-1}$. (c) Experimental near-field result of a triangular-shaped micro antenna with an inner angle $\delta = 33^{\circ}$ and a scanning area of 70 × 50 μm. (d) Line cut profile with an angle of 25° w.r.t the x axis for the triangle-shaped micro antenna in c. green balls represent the actual line cut profile data and the dark green line is the sine damp fitted function (equation 21) with a decay constant of $1.22 \times 10^4\ m^{-1}$. (e) Experimental near-field result of a triangular-shaped micro antenna with an inner angle $\delta = 43^{\circ}$ and a scanning area of 70 × 50 μm. (f) Line cut profile with an angle of 25° w.r.t the x axis for the triangle-shaped micro antenna in e. red balls represent the

actual line cut profile data and the Brown is the sine damp fitted function (equation 21) with a decay constant of $3.0341 \times 10^4\ m^{-1}$.

References

1. Ma, W., Hu, G., Hu, D., Chen, R., Sun, T., Zhang, X., Dai, Q., Zeng, Y., Alù, A., & Qiu, C.-W.(s). Ghost hyperbolic surface polaritons in bulk anisotropic crystals. Nature Name 2021, 596 (7872), 362-366.
2. Babar, S., & Weaver, J.(s). Optical constants of Cu, Ag, and Au revisited. Applied Optics Name 2015, 54 (3), 477-481.
3. Hu, C., Sun, T., Zeng, Y., Ma, W., Dai, Z., Yang, X., Zhang, X., & Li, P.(s). Source-configured symmetry-broken hyperbolic polaritons. eLight Name 2023, 3 (1), 14.
4. Matson, J., Wasserroth, S., Ni, X., Obst, M., Diaz-Granados, K., Carini, G., Renzi, E. M., Galiffi, E., Folland, T. G., & Eng, L. M.(s). Controlling the propagation asymmetry of hyperbolic shear polaritons in beta-gallium oxide. Nature Communications Name 2023, 14 (1), 5240.
5. Lv, J., Wu, Y., Liu, J., Gong, Y., Si, G., Hu, G., Zhang, Q., Zhang, Y., Tang, J.-X., & Fuhrer, M. S.(s). Hyperbolic polaritonic crystals with configurable low-symmetry Bloch modes. Nature communications Name 2023, 14 (1), 3894.
6. Álvarez-Pérez, G., Duan, J., Taboada-Gutiérrez, J., Ou, Q., Nikulina, E., Liu, S., Edgar, J. H., Bao, Q., Giannini, V., & Hillenbrand, R.(s). Negative reflection of nanoscale-confined polaritons in a low-loss natural medium. Science advances Name 2022, 8 (29), eabp8486.
7. Ying, L., Zhou, M., Mattei, M., Liu, B., Campagnola, P., Goldsmith, R. H., & Yu, Z.(s). Extended range of dipole-dipole interactions in periodically structured photonic media. Physical Review Letters Name 2019, 123 (17), 173901.
